# Cell‐free and extracellular vesicle microRNAs with clinical utility for solid tumors

**DOI:** 10.1002/1878-0261.13709

**Published:** 2024-08-11

**Authors:** Yoshinori Hayashi, Janelle‐Cheri Millen, Romela Irene Ramos, Jennifer A. Linehan, Timothy G. Wilson, Dave S. B. Hoon, Matias A. Bustos

**Affiliations:** ^1^ Department of Translational Molecular Medicine Saint John's Cancer Institute at Providence Saint John's Health Center Santa Monica CA USA; ^2^ Department of Surgical Oncology Saint John's Cancer Institute at Providence Saint John's Health Center Santa Monica CA USA; ^3^ Department of Urology and Urologic Oncology Saint John's Cancer Institute at Providence Saint John's Health Center Santa Monica CA USA; ^4^ Department of Genome Sequencing Center Saint John's Cancer Institute at Providence Saint John's Health Center Santa Monica CA USA

**Keywords:** cfmiRs, diagnostic, extracellular vesicles, fluid molecular biopsy, prognostic, solid tumors

## Abstract

As cutting‐edge technologies applied for the study of body fluid molecular biomarkers are continuously evolving, clinical applications of these biomarkers improve. Diverse forms of circulating molecular biomarkers have been described, including cell‐free DNA (cfDNA), circulating tumor cells (CTCs), and cell‐free microRNAs (cfmiRs), although unresolved issues remain in their applicability, specificity, sensitivity, and reproducibility. Translational studies demonstrating the clinical utility and importance of cfmiRs in multiple cancers have significantly increased. This review aims to summarize the last 5 years of translational cancer research in the field of cfmiRs and their potential clinical applications to diagnosis, prognosis, and monitoring disease recurrence or treatment responses with a focus on solid tumors. PubMed was utilized for the literature search, following rigorous exclusion criteria for studies based on tumor types, patient sample size, and clinical applications. A total of 136 studies on cfmiRs in different solid tumors were identified and divided based on tumor types, organ sites, number of cfmiRs found, methodology, and types of biofluids analyzed. This comprehensive review emphasizes clinical applications of cfmiRs and summarizes underserved areas where more research and validations are needed.

## Graphical abstract

Cell‐free microRNAs (cfmiRs) are small‐RNA circulating molecules detectable in almost all body biofluids. Innovative technologies have improved the application of cfmiRs to oncology, with a focus on clinical needs for different solid tumors, but with emphasis on diagnosis, prognosis, cancer recurrence, as well as treatment monitoring. This comprehensive review summarizes the clinical applications of cfmiRs and current underserved research areas.

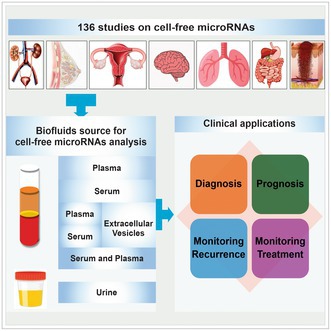

**Abbreviations**
AUC: area under the curve
BC: breast cancer
BLCA: bladder cancer
CA125: carbohydrate antigen125
CA19‐9: carbohydrate antigen 19‐9
CC: cholangiocarcinoma
CEA: carcinoembryonic antigen
CeC: cervical cancer
cfDNA: cell‐free DNA
cfmiRs: cell‐free microRNAs
cfNA: cell‐free nucleic acid
CM: cutaneous melanoma
CRC: colorectal cancer
CRT: chemoradiotherapy
CSF: cerebral spinal fluid
CT: computed tomography
CTCs: circulating tumor cells
ddPCR: droplet digital polymerase chain reaction
DFS: disease‐free survival
EC: esophageal cancer
EnC: endometrial cancer
EOCRC: early‐onset colorectal cancer
EV: extracellular vesicle
GBC: gallbladder cancer
GBM: glioblastoma
GC: gastric cancer
GL: glioma
HCC: hepatocellular carcinoma
HD: healthy donors
HPV: human papillomavirus
HR: hazard ratio
ICI: immune checkpoint inhibitor
LC: lung cancer
MBM: melanoma brain metastasis
miR: microRNA
mRNA: messenger RNA
mtDNA: mitochondrial DNA
ncRNA: non‐coding RNA
NGS: next‐generation sequencing
NIH: National Institutes of Health
NSCLC: non‐small cell lung cancer
OC: ovarian cancer
OS: overall survival
PC: pancreatic cancer
PCR: polymerase chain reaction
PD‐1: programed cell death protein 1
PD‐L1: programmed cell death ligand‐1
PFS: progression‐free survival
PRCA: prostate cancer
PSA: prostate specific antigen
qRT‐PCR: quantitative reverse transcription polymerase chain reaction
RCC: renal cell carcinoma
SPN: solitary pulmonary nodule
TME: tumor microenvironment

## 1 Introduction

Cell‐free nucleic acids (cfNAs) were first described in 1948 by Mandel and Metais [1]. However, several decades passed with minimal clinical applications of this knowledge until mutated fragments of the *RAS* gene were detected in the blood of cancer patients in 1994 [2, 3], and not long thereafter genomic alterations in cfDNA [4, 5, 6]. This sparked interest in the potential use of cfNAs as ‘liquid biopsy’ biomarkers in noninvasive cancer assays [7]. Liquid biopsy terminology is always linked to the study of cfNAs and circulating tumor cells (CTCs). Therefore, liquid biopsy is misleading, as it refers to blood‐based biomarkers and it does not consider other types of body fluids. Cancer blood biomarkers can cover many different analytes that include proteins, lipids, and small molecules (non‐nucleic acids), whereby cfNA is only one type of blood biomarker. Therefore, in this review we will refer to liquid biopsy as ‘biofluid molecular biopsy.’

CfNAs enable the assessment and monitoring of cancer patients during the course of the disease at any point in time by sampling a biofluid using various molecular approaches. This type of fluid molecular biopsy could potentially reflect molecular changes that otherwise cannot be assessed by imaging technologies or minimally invasive techniques, such as tumor biopsy sampling. Concurrent with these developments in oncology, cfNAs were being used in fetal medicine, wherein circulating fetal DNA was identified in maternal serum and applied successfully to the prenatal diagnosis of chromosomal disorders [8]. The more recent applications in the last decade were fueled by polymerase chain reaction (PCR) and then next‐generation sequencing (NGS) in combination with different technologies, expanding the utility and clinical applications of the different types of cfNAs to several cancer types [9, 10].

The levels of DNA, mRNA, microRNA (miR), mitochondrial DNA (mtDNA), and noncoding RNA (ncRNA) in biofluids reflect pathologic processes such as cancer, inflammatory disorders, trauma, stroke, cardiovascular disease, sepsis, and other clinical conditions [9, 10, 11]. MiRs are a family of short noncoding single‐stranded nucleic acids (18–22 nucleotides in size) that can repress the translation or alternately degrade specific target mRNAs by complementary binding, presumed to regulate greater than 50% of protein‐coding genes at the post‐transcriptional level [12]. The changes in miR profiles in tumor cells can disrupt crucial signaling pathways of major oncogenes and tumor suppressor genes, potentially promoting various aspects of tumor proliferation and metastasis [13, 14]. MiRs in cancer cells are functional regulatory molecules recognized as a hallmark of cancer [15]. CfmiRs, released from cancer cells into circulation, are stable and abundantly detected in various body fluids [16]. Given the inherent biological function and structure, cfmiRs offer significant advantages as biomarkers for the detection of cancer in biofluids. This review updates the current state of circulating cfmiRs found in plasma, serum, and urine of patients diagnosed with different types of solid tumors. Also, the review emphasizes cfmiRs potential for clinical applications in diagnostics, prognostication, disease monitoring, and evaluating responses to various therapies.

## 2 Types of Circulating miRs

The miRs play not only a role in the intracellular environment but also the extracellular environment of the source cells [17]. Extracellular miRs are released as direct active secretion or passive leakage at the time of cell death, such as through apoptosis, physical cell disruption, or necrosis [18, 19]. Extracellular miRs were once thought of as waste products of cellular metabolism, but extracellular miRs mediate several functions during intercellular communication between cancer cells and the tumor microenvironment (TME) among other functions during the different steps of the metastatic cascade [20].

CfmiRs exist in extracellular environments in a variety of forms, such as free‐floating complex forms with binding proteins, as cargoes of extracellular vesicles (EVs) of different sizes, and in association with nonvesicular nanoparticles [21, 22]. The complex forms between miRs and proteins as well as the association of miRs EVs or with nonvesicular nanoparticles have been previously reviewed [22]. EVs protect miRs from degradation by endogenous ribonucleases, making them even more stable in the extracellular fluid environment [23]. Several biological mechanisms have been proposed for EVs, as discussed in previous reviews [22, 24], such as incorporation into recipient cells. The miRs present in EVs mediate communication between cells and regulate the mRNA expression in recipient cells as our and other groups have shown [25, 26]. Therefore, miRs as cargo in EVs represent alternative analytes for clinical biomarkers development in solid tumors.

## 3 Summary of cfmiR Studies in Solid Tumors

**Fig. 1:**
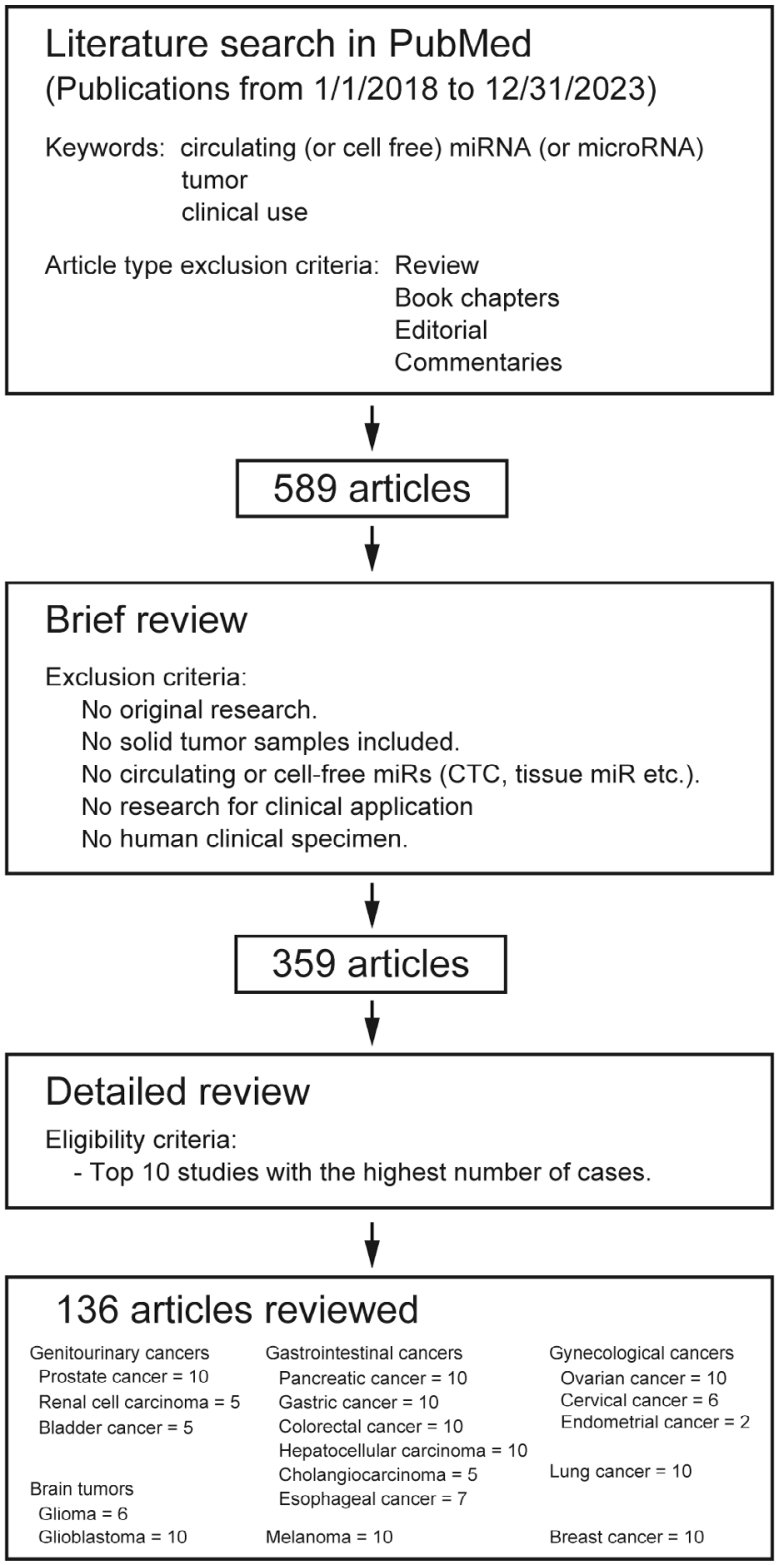
Inclusion criteria for the cfmiR studies selected and discussed in this review.

**Fig. 2:**
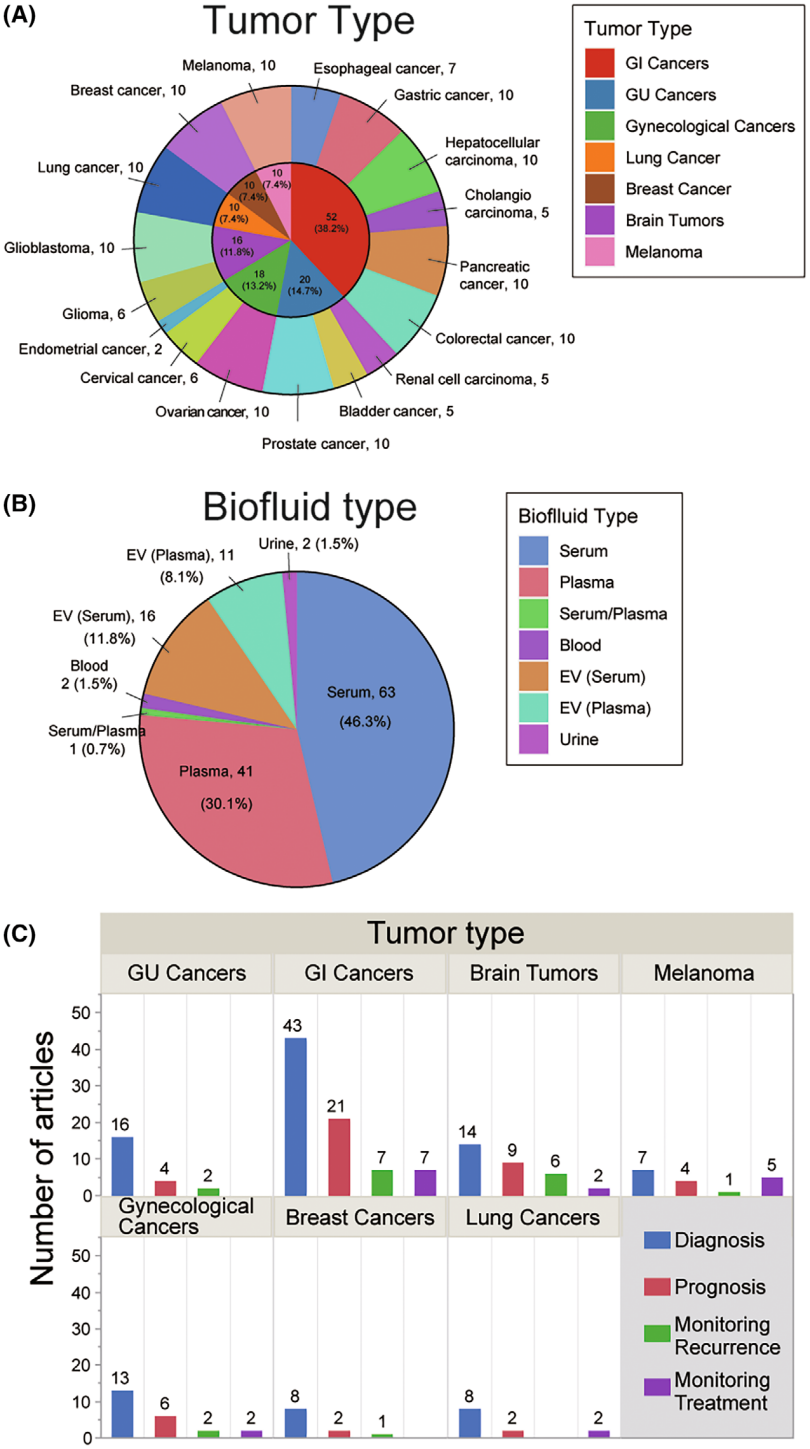
Clinical applications of cfmiRs in solid tumors. (A) Summary of the tumor types included in the review. (B) Pie graph showing the distribution of the articles based on the types of biofluid analyzed. (C) Number of articles distributed across the tumor types and the clinical applications.

In this review we narrowed down the focus to 136 cancer‐related articles through a literature search and detailed review, as shown in Fig. 1. The bibliography search in the PubMed database was performed using keywords such as ‘circulating (or cell‐free) miRNA (or microRNA),’ ‘tumor,’ and ‘clinical use.’ Review articles, book chapters, editorials, or commentaries were excluded to obtain 589 articles. Subsequently, articles were excluded if they did not contain original research, or if the focus was on the nonsolid tumors, not concerning circulating or cell‐free miRs, nonhuman subjects, or with nonclinical purposes. Of the 359 studies filtered, only the 10 studies with the highest number of cases or all studies available for each tumor type were selected to lessen the search to 136 articles. The 136 studies were categorized based on the organ site of development/metastasis, tumor type, type of biofluid analyzed, and significant clinical correlations (Fig. 2A). The majority of the studies were focused on serum (46.3%), followed by plasma (29.4%), although the assessment of cfmiRs in urine has been recently applied to investigate genitourinary cancers (Fig. 2B). There were also 27 studies focused on EVs containing miRs isolated from serum and plasma (Fig. 2B), and this is a field that has exponentially grown over the past years.

## 4 Clinical Utility of miRs in Solid Tumors

The main clinical applications for cfmiR reside around diagnostic and prognostic utility. The standard approach to the diagnosis of cancer involves obtaining a tumor tissue biopsy. However, depending on the tumor type and the anatomic location, invasive procedures are frequently required. The potential morbidity of an invasive procedure is sometimes coupled with the technical complexity of the procedure, which may result in low diagnostic sensitivity. In many cancers, such as brain tumors, breast cancer (BC), prostate cancer (PRCA), colorectal cancers (CRC), and other solid tumors, the current diagnostic methods are invasive. Many solid tumors, such as pancreas‐ and hepatobiliary‐related cancers are diagnosed at a late stage when symptoms are developed, and this later stage has been associated with a worse prognosis. Early diagnosis and prognostic prediction of cancer using noninvasive methods remains both a goal and challenge for most solid tumors. Fluid molecular biopsy utilizing the features of cfmiRs is an attractive potential method, which avoids invasive procedures, enabling earlier diagnosis and accurate analytic interpretations of patients’ cancer clinical status.

Another area of interest for cfmiR biofluid biopsy‐based approaches is in monitoring tumor recurrence or treatment responses. After a diagnosis, cancer patients often need multiple tests over an extended period, both before and after treatment, to conduct surveillance for recurrence, or to assess treatment response. As mentioned earlier, tissue biopsies face several barriers for multiple invasive attempts. Therefore, there is significant anticipation for the potential of less invasive and highly accurate repetitive fluid‐based tests, capturing the characteristics of cfmiRs generated from specific cancer types and their alterations in response to treatments.

In recent years, the research on cfmiRs has focused not only on individual cfmiRs, but more on the combination of multiple cfmiRs, referred to as panels or signatures. Single biomarkers in the blood are risky in reproducible accuracy and sensitivity, particularly due to cancer heterogeneity and evolving changes in tumor composition during progression.

**Fig. 3:**
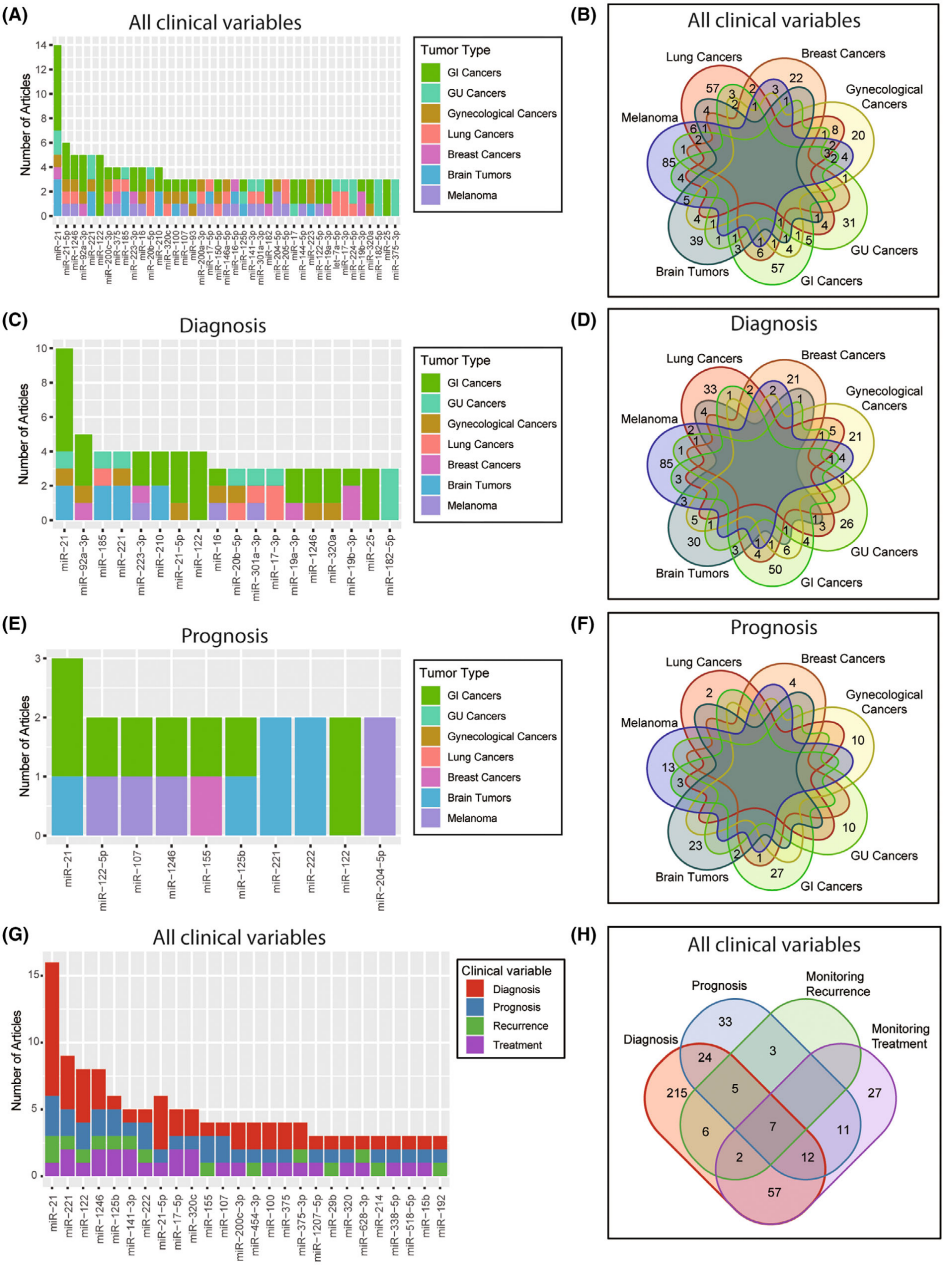
Summary of the cfmiRs found in all the articles reviewed. (A) Number of articles separated based on the tumor type. (B) Venn diagram showing the overlapping cfmiRs in all the articles reviewed. (C) Number of articles assessing diagnostic cfmiRs separated based on the tumor types. (D) Venn diagram showing the overlapping cfmiRs with diagnostic utility in all the articles reviewed. (E) Number of articles assessing prognostic cfmiRs separated based on the tumor types. (F) Venn diagram showing the overlapping cfmiRs with prognostic utility in all the articles reviewed. (G) Number of articles assessing cfmiRs applied to all clinical variables and separated based on the tumor types. (H) Venn diagram showing the overlapping cfmiRs across all clinical variables in all the articles reviewed.

In this review, rigorous literature searches were performed to summarize previous clinical application of cfmiRs as potential biomarkers. Of the 136 studies identified, 109 were related to diagnosis, 48 were focused on prognosis, and the rest were associated with monitoring disease recurrence or treatment responses, although some studies overlapped in these categories (Fig. 2C). The studies reviewed were selected on specific criteria, as described above, and most importantly, clinical correlations with adequate sample size. Also, cfmiRs identified throughout the articles included in this review were classified based on potential for clinical applications: diagnostic, prognostic, and monitoring treatment/recurrence (Fig. 3A). Then the overlapped cfmiRs were identified among the tumor type categories (Fig. 3B). miR‐21 was the most‐studied cfmiR and exhibited a clinical value in 14 studies across five tumor type categories. As a diagnostic marker, miR‐21 exhibited an area under the curve (AUC) that ranged from 0.618 to 0.999 across different solid tumors, with glioblastoma (GBM) having the best performance (Table 1). Additionally, cfmiRs were divided based on the diagnostic (Fig. 3C,D) or prognostic (Fig. 3E,F) utility across all the tumor types analyzed. Furthermore, the overlapped cfmiRs were categorized into the four clinical variable groups: diagnostic, prognostic, tumor recurrence, and treatment (Fig. 3G,H **)**. Seven miRs (miR‐21, miR‐221, miR‐122, miR‐1246, miR‐125b, miR‐141‐3p, and miR‐222) were commonly found to have a potential application for diagnosis, prognosis, monitoring recurrence, and monitoring treatment (Fig. 3H). The identification of overlapping miRs across different tumor types suggested a potential interference affecting the specificity in clinical applications.

**Table 1:** Summary of the commonly reported cfmiRs with potential diagnostic utility across different tumor types.

Target	Tumor type	Signature	AUC	*P* value	Sensitivity (%)	Specificity (%)	PMID
miR‐21, miR‐21‐3p, miR‐21‐5p							
miR‐21	OC	Single	0.740	0.0001	61	82	30107086
miR‐21	GBC	Single	0.80	< 0.0001	75	83.6	36682281
miR‐21	GBM	Single	0.999	< 0.0001	100	95	32316783
miR‐21	GL	Single	0.618	0.0014	–	–	32915353
miR‐21	PRCA	Single	0.959	< 0.0001	90.9	100	31483058
miR‐21	CC	Single	0.785	< 0.001	72.9	70.6	33805513
miR‐21	PC	Single	0.869	–	80	90	32724386
miR‐21	ESCC	Single	0.928	–	88.3	97.3	32021418
miR‐21	ESCC	Panel	–	–	–	–	35148754
miR‐21	ESCC	Panel	–	–	–	–	34799871
miR‐21‐5p	PC	Single	0.86	–	82	78	31009404
miR‐21‐5p	CRC	Panel	–	–	–	–	34376648
miR21‐5p	CeC	Panel	–	–	–	–	31658043
miR‐21‐3p	LC	Panel	–	–	–	–	32134442
miR‐122, miR‐122‐5p							
miR‐122	HCC	Single	0.82	–	–	–	32424223
miR‐122	HCC	Single	0.759	< 0.001	83	64	35666610
miR‐122	CC	Single	0.992	–	94.9	100	30653586
miR‐122	CC	Single	0.707	< 0.001	53.5	80.9	33805513
miR‐122‐5p	HCC	Single	0.741	< 0.001	76.4	68.9	34887498
miR‐221							
miR‐221	GBM	Single	0.925	–	90	100	31422498
miR‐221	GBM	Single	0.956	–	88	100	32989634
miR‐221	PRCA	Single	0.872	< 0.0001	81.8	72.5	31483058
miR‐221	CeC	Panel	–	–	–	–	30254211
miR‐92a, miR‐92a‐3p							
miR‐92a‐3p	EAC	Single	0.76	0.014	58	85	32131978
miR‐92a‐3p	GC	Single	0.829	0.026	65.6	87.7	31009404
miR‐92a‐3p	BC	Panel	–	–	–	–	32894240
miR‐92a‐3p	CRC	Panel	–	–	–	–	34376648
miR‐92a‐3p	OC	Panel	–	–	–	–	35530324
miR‐185							
miR‐185	PRCA	Single	0.945	–	–	–	35835374
miR‐185	NSCLC	Single	0.790	–	68	78.7	33251978
miR‐223, miR‐223‐3p							
miR‐223	GL	Single	0.777	< 0.0001	–	–	32915353
miR‐223	OC	Panel					30107086
miR‐223‐3p	UVM	Single	0.95	< 0.0001	80	92	36619870
miR‐223‐3p	BC	Panel	–	–	–	–	32894240
miR‐223‐3p	PC	Panel	–	–	–	–	31006985
miR‐210							
miR‐210	PC	Single	0.823	–	83	90	32724386
miR‐210	CRC	Panel		–	–	–	35850198

AUC, area under the curve; BC, breast cancer; CC, colon cancer; CeC, cervical cancer; cfmiR, cell‐free miR; CRC, colorectal cancer; EAC, esophageal adenocarcinoma; ESCC, esophageal squamous cell carcinoma; GBM, glioblastoma; GC, gastric cancer; GL, glioma; HCC, hepatocellular carcinoma; LC, lung cancer; NSCLC, non‐small cell lung cancer; OC, ovarian cancer; PC, pancreatic cancer; PMID, PubMed identification; PRCA, prostate cancer; UVM, uveal melanoma.

### 4.1 Genitourinary cancers

#### 4.1.1 Prostate cancer

PRCA is the most frequently diagnosed cancer among men in the USA and it ranks as the third leading cause of cancer‐related death in men [27]. Diagnosis of PRCA is performed by a combination of digital rectal examination, prostate‐specific antigen (PSA), and prostate tissue biopsy. The main problem is that PSA still has low specificity and does not distinguish between latent and aggressive forms of the disease [28]. In addition, the high false‐positive rates of PSA often lead to unnecessary biopsies [29]. Although PRCA tissue biopsies have improved, the process remains invasive.

To improve the diagnosis of PRCA, Urabe *et al*. [30] conducted a comprehensive miR profiling on serum samples from 809 PRCA patients, 741 biopsy‐negative patients, and 41 healthy donors (HD) using microarray analysis. All serum samples were divided into a discovery, a training, and a validation cohort. The authors identified that the optimal combination of two miRs (miR‐17‐3p and miR‐1185‐2‐3p) had high diagnostic performance (AUC = 0.95, sensitivity = 0.90, specificity = 0.90). In a study conducted by Mello‐Grand *et al*. [31], two diagnostic miRs (miR‐103a‐3p and let‐7a‐5p) were selected from plasma samples obtained from 60 PRCA patients. The classification model combining these two miRs with PSA demonstrated an AUC value of 0.76 in the overall cohort and an AUC of 0.86 in a subgroup of patients with PSA levels below 4 ng·mL^−1^. In an independent validation cohort, miR‐103a‐3p or let‐7a‐5p combined with PSA showed better diagnostic performance compared to PSA alone (AUCs = 0.74 and 0.79, respectively).

The identification of prognostic factors for PRCA patients is a clinical need. Takamori *et al*. [32] investigated the prognostic utility for PRCA of cfmiRs using the miR microarray assay. The authors optimized a prognostic prediction model based on the detection levels of three miRs (miR‐3147, miR‐4513, and miR‐4728‐5p) in pretreatment serum samples from 295 PRCA patients who underwent radical prostatectomy, along with the pathological T stage and Gleason score of the excised specimens. The model distinguished PRCA patients who had shorter biochemical recurrence relapse‐free survival (AUC = 0.80, *P* < 0.001); however, each cfmiRs showed low performance, suggesting that T stage and Gleason score may drive the proposed model.

In summary, these studies suggest the potential diagnostic and prognostic applications of cfmiRs in PRCA, although there is inconsistency in overlapping miRs (except for miR‐21, miR‐121, and miR‐141; Table 2). There is also an overall low performance of cfmiRs in diagnostic and prognostic studies for PRCA. Overall, the research on cfmiRs in PRCA highlights the large number of patients included in the studies, but the need for better‐defined larger cohorts of PRCA patients. More robust cfmiRs biomarkers are needed to improve cancer diagnosis without relying on PSA or invasive tissue biopsies only, to reduce the number of surgical procedures, and to enhance predictions of aggressive PRCA tumors. Also, there is a major need in the context of metastatic castration‐resistant patients under treatment in monitoring disease recurrence and treatment responses.

**Table 2:** Summary of the cfmiRs with potential clinical utility for genitourinary cancers.

Tumor type	Technology	cfmiR signature		cfmiR status	Analyte	Patients (*N*)	Control group (*N*)	Clinical variable	Author (year)	PMID
PRCA	Microarray	Panel	[miR‐17‐3p, miR‐1185‐2‐3p]	–	Serum	809	HD (41); Negative cohort with prostate biopsy (741)	Diagnosis	Urabe F *et al*. (2019)	30808771
PRCA	Microarray	Panel	[miR‐3147, miR‐4513, miR‐4728‐5p]	–	Serum	295	None	Prognosis (DFS)	Takamori H *et al*. (2022)	35971801
PRCA	qRT‐PCR	Single	miR‐93; miR‐221; miR‐125b; miR‐21	Increase	Plasma	149	None	Recurrence	Zedan AH *et al*. (2019)	30537232
PRCA	qRT‐PCR	Panel	[miR‐103a‐3p, let‐7a‐5p]	–	Plasma	128	HD (82); Other prostate disease (144)	Diagnosis	Mello‐Grand M *et al*. (2019)	30452625
PRCA	qRT‐PCR	Panel	[miR‐24, miR‐30c]	–	Urine	103		Diagnosis	Zhao F *et al*. (2019)	30777394
PRCA	qRT‐PCR	Panel	[miR‐145, miR‐148, miR‐185]	–	Plasma	92	Precancerous lesions (26); hyperplasia (49)	Diagnosis	Coradduzza D *et al*. (2022)	35835374
PRCA	qRT‐PCR	Single	miR‐494	Increase	Serum	90	HD (90); Benign hyperplasia (90)	Diagnosis	Cai B *et al*. (2019)	31414754
PRCA	qRT‐PCR	Single	miR‐141‐3p; miR‐375‐3p; miR‐221‐3p	Increase	Plasma	84	None	Prognosis (OS), Recurrence	Zedan AH *et al*. (2020)	31937854
PRCA	qRT‐PCR	Panel	[miR‐18a, miR‐21, miR‐221, miR‐141]	–	Plasma	80	Benign hyperplasia (30)	Diagnosis	Ibrahim NH *et al*. (2019)	31483058
PRCA	NGS‐based	Panel	[miR‐17‐5p, miR‐106a‐5p]; [miR‐20a‐5p, miR‐20b‐5p]	–	Serum	78	None	Prognosis (Risk classification)	Hoey C *et al*. (2019)	31122242
RCC	qRT‐PCR	Panel	[miR‐224‐5p, miR‐34b‐3p, miR‐182‐5p]	–	Serum	146	HD (150)	Diagnosis	Huang G *et al*. (2020)	32556891
RCC	ddPCR	Panel	[miR‐182‐5p, miR‐375‐3p]	–	Plasma	119	HD (74)	Diagnosis	Sequeira JP *et al*. (2023)	37762193
RCC	qRT‐PCR	Single, Panel	miR‐155‐5p; [miR‐1‐3p, miR‐155‐5p, miR‐200b‐3p, miR‐224‐5p]	–	Serum	112	None; HD (112)	Prognosis (OS); Diagnosis	Li R *et al*. (2023)	36727070
RCC	qRT‐PCR	Panel	[miR‐20b‐5p, miR‐30a‐5p, miR‐196a‐5p]	–	Serum	110	HD (110)	Diagnosis	Huang G *et al*. (2020)	32823234
RCC	qRT‐PCR	Panel	[miR‐508‐3p, miR‐885‐5p]	–	Serum	85	HD (35)	Diagnosis	Liu S *et al*. (2019)	31661117
BLCA	Microarray	Single‐Panel	miR‐6087; [miR‐6087, miR‐6724‐5p, miR‐3960, miR‐1343‐5p, miR‐1185‐1‐3p, miR‐6831‐5p, miR‐4695‐5p]; [miR‐6087, miR‐1343‐5p]	Decrease	Serum	392	HD (100); Other cancers (480)	Diagnosis	Usuba W *et al*. (2019)	30382619
BLCA	qRT‐PCR	Panel	[miR‐130a‐3p, miR‐130b‐3p, miR‐301a‐3p]	–	Serum	74	HD (90)	Diagnosis	Wang J *et al*. (2020)	32761678
BLCA	ddPCR	Panel	[miR‐182‐5p, miR‐375‐3p]	–	Plasma	73	HD (74)	Diagnosis	Sequeira JP *et al*. (2023)	37762193
BLCA	qRT‐PCR	Single	miR‐663b	Increase	EV (Plasma)	63	HD (59)	Diagnosis	Yin X *et al*. (2020)	31872468
BLCA	qRT‐PCR	Single	miR‐203	–	Urine	25	HD (25); Benign hyperplasia (10)	Diagnosis	Singh P *et al*. (2022)	35445913

BLCA, bladder cancer; cfmiR, cell‐free miR; ddPCR, droplet digital polymerase chain reaction; DFS, disease‐free survival; EV, extracellular vesicles; HD, healthy donor; NGS, next‐generation sequencing; OS, overall survival; PMID, PubMed identification; PRCA, prostate cancer; qRT‐PCR, quantitative reverse transcription polymerase chain reaction; RCC, renal cell carcinoma.

#### 4.1.2 Renal cell carcinoma

Renal cell carcinoma (RCC) represents 80–90% of malignant tumors in the kidney and accounts for 3% of all cancers [27, 33]. Most RCC patients remain asymptomatic until the advanced stage, making early detection crucial for deciding the treatment strategies and patient prognosis. Li *et al*. [34] measured the levels of potential diagnostic cfmiRs in serum samples from 112 RCC patients and 112 HD. The authors constructed a diagnostic model based on an optimized panel of four miRs (miR‐1‐3p, miR‐155‐5p, miR‐200b‐3p, and miR‐224‐5p), which demonstrated an AUC of 0.903. Additionally, the elevated levels of miR‐155‐5p were associated with poor prognosis (*P* = 0.0017). Huang *et al*. [35] measured the serum cfmiR levels using qRT‐PCR in a total of 146 RCC patients and 150 HD, selecting cfmiR candidates through a three‐stage process of screening, testing, and validation. The panel composed of three miRs (miR‐224‐5p, miR‐34b‐3p, and miR‐182‐5p) demonstrated an AUC of 0.855.

The five cfmiR studies were applied to the diagnosis of RCC patients and all of them employed PCR‐based methods (four using qRT‐PCR and one using ddPCR; Table 2) [36, 37, 38]. In the five RCC studies, the target selection was based on previous public data or literature review. Although previous studies characterized miR signatures of the different RCC histological subtypes, those signatures have not been validated in biofluids. In that regard, our group has recently profiled a small cohort of RCC patients using paired urine and plasma samples [39]. In addition, cfmiR applications to prognosis as well as monitoring recurrence or treatment should be a priority for RCC patients. Future comprehensive validation analyses using high‐throughput NGS‐based assays are needed, especially in the context of metastatic RCC setting.

#### 4.1.3 Bladder cancer

Bladder cancer (BLCA) is one of the most common malignant tumors in the genitourinary system and ranks as the tenth leading cause of cancer‐related deaths worldwide [27]. While the 5‐year overall survival (OS) rate for BLCA exceeds 70%, it significantly decreases to ~10–30% when it develops into metastatic disease [40]. The prognosis of BLCA significantly worsens as it progresses to the advanced stage, and therefore, early diagnosis is crucial to improve outcomes. Usuba *et al*. [41] analyzed cfmiR profiles from 972 serum samples, including 392 cases of BLCA, 480 cases of other cancers, and 100 noncancer controls using a microarray assay. Both the levels of miR‐6087 (AUC = 0.89) as a single biomarker and a diagnostic model based on seven miRs (miR‐6087, miR‐6724‐5p, miR‐3960, miR‐1343‐5p, mIR‐1185‐1‐3p, miR‐6831‐5p, and miR‐4695‐5p; AUC = 0.97) showed significant potential for classifying patients with BLCA.

The miR‐130 family was investigated by Wang *et al*. [42] to develop early diagnostic biomarkers for BLCA. PCR‐based miR profiling was conducted in plasma samples from 74 BLCA patients and 90 HD to develop a 3‐miR panel (miR‐130a‐3p, miR‐130b‐3p, and miR‐301a‐3p) that demonstrated an AUC of 0.961. The rest of the reports on the clinical role of cfmiRs in genitourinary cancer are summarized in Table 2 [36, 37, 38, 43, 44, 45, 46, 47, 48, 49, 50, 51]. These studies suggest that blood and urine fluids may have potential clinical utility for the diagnosis of BLCA. More importantly, monitoring of early‐stage BLCA identifying early‐stage treatment such as BCG resistance is needed. Also, another problem is that there is a lack of studies focused on cfmiRs predicting prognosis, monitoring recurrence, and monitoring treatment. Therefore, more research should be focused on these clinical applications to explore and expand the utility of cfmiRs in BLCA.

### 4.2 Gastrointestinal cancers

#### 4.2.1 Colorectal cancer

CRC is the third most common cancer incidence and remains the second‐leading cause of cancer‐related deaths worldwide [27]. Seven major studies have focused on the potential for significant differences in cfmiR levels in biofluids of CRC patients compared to healthy individuals at the time of diagnosis, highlighting the application of cfmiRs for early detection [52, 53, 54, 55, 56, 57, 58]. Raut *et al*. [54] profiled 41 cfmiRs in the serum of a total of 376 individuals, including 198 who developed CRC, during a follow‐up period of over 10 years. The authors created a score based on seven miRs (let‐7g‐5p, miR‐19a‐3p, miR‐23a‐3p, miR‐92a‐3p, miR‐144‐5p, miR‐21‐5p, and miR‐27a‐3p) with potential diagnostic value, demonstrating a higher diagnostic predictive ability than existing risk scores (AUC = 0.802). Nakamura *et al*. [53] created a 4‐miR panel to identify patients with early‐onset CRC (EOCRC) and demonstrated high diagnostic efficacy for EOCRC through training and validation using plasma samples from two independent cohorts (AUC = 0.92 and 0.88, respectively). Shibamoto *et al*. [59] conducted a microarray‐based assay to examine 32 miRs on plasma samples collected from CRC patients before and after curative resection, which aimed to explore the role of cfmiRs in prognosis and recurrence monitoring. High levels of miR‐4442 before surgery were associated with recurrence and were identified as an independent factor for poor recurrence‐free survival (hazard ratio [HR] = 2.074, *P* = 0.012). Other prognostic cfmiR candidates reported include miR‐21, miR‐30a‐5p, miR‐320c, and miR‐98 [55, 57, 58, 60]. Han *et al*. [61] analyzed 646 cfmiRs using microarray technology on plasma samples from CRC patients who underwent oxaliplatin‐based chemotherapy for treatment monitoring. A 6‐miR panel (miR‐100, miR‐92a, miR‐16, miR‐30e, miR‐144‐5p, and let‐7i) distinguished CRC patients resistant to chemotherapy.

CfmiRs are emerging as an alternative tool, since cfDNA has not shown clinical applicability for detecting the early stages of CRCs. The studies on CRC included in this review involved validation in large cohorts exceeding 100 patients, and many clinical applications across the four categories defined. A need for CRC patients is to define cfmiRs that are linked to aggressive types of disease as well as early‐stage diagnosis in conjunction with imaging‐based approaches.

#### 4.2.2 Esophageal cancers

Esophageal cancer (EC) is the sixth most common cause of cancer‐related death worldwide, accounting for just over half a million deaths annually [27]. EC has two major histological subtypes: adenocarcinoma (EAC) and squamous cell carcinoma (ESCC) [62]. Among the articles reviewed, two publications focused on EAC, four articles targeted ESCC, and one article combined both EAC and ESCC. The prognosis for patients who develop EC remains poor, with 5‐year OS rates ranging from 12% to 20% [62]. Diagnostic cfmiRs were evaluated by Miyoshi *et al*. [63] using serum samples from a cohort of 737 ESCC patients. The authors investigated the detection of miRs in serum, based on miRs that exhibited characteristic features at the tissue level, and created an 8‐miR panel (miR‐103, miR‐106b, miR‐151, miR‐17, miR‐181a, miR‐21, miR‐25, miR‐93). This panel was then validated in three large independent cohorts, demonstrating high diagnostic performance (AUC = 0.80; AUC = 0.89; and AUC = 0.92, respectively). Importantly, the 8‐miR panel outperformed common clinical serum markers of squamous cell carcinoma antigen and carcinoembryonic antigen (CEA, *P* < 0.001).

Han *et al*. [64] demonstrated the prognostic utility of miR‐338‐5p in EC. The levels of miR‐338‐5p were measured in the serum of 104 patients with ESCC who underwent neoadjuvant chemoradiotherapy (CRT). High levels of miR‐338‐5p were associated with longer OS. Additionally, serum miR‐338‐5p levels correlated with pathologic treatment response to CRT. Other cfmiRs (miR‐34a, miR‐21, miR‐92‐3p, and miR‐375, etc.) have been considered for clinical applications in EC [65, 66, 67, 68, 69] and are summarized in Table 3.

**Table 3:** Summary of the cfmiRs with potential clinical utility for gastrointestinal cancers.

Tumor type	Technology	cfmiR signature		cfmiR status	Analyte	Patients (*N*)	Control group (*N*)	Clinical variable	Author (year)	PMID
ESCC	qRT‐PCR	Panel	[miR‐103, miR‐106b, miR‐151, miR‐17, miR‐181a, miR‐21, miR‐25, miR‐93]	Increase	Serum	737	HD (435)	Diagnosis	Miyoshi J *et al*. (2022)	35148754
ESCC	qRT‐PCR	Single	miR‐338‐5p	Decrease	Serum	104	HD (50)	Diagnosis, Prognosis (OS), Treatment (5FU + cisplatin [Neoadjuvant])	Han L *et al*. (2019)	31646712
EC	qRT‐PCR	Single	miR‐34a	Increase	Plasma	101	HD (97); Benign disease (31)	Diagnosis	Lin Y *et al*. (2019)	31710448
Esophagogastric cancer	ddPCR	Single	miR‐375	Decrease	Plasma	68	None	Prognosis (OS, DFS)	van Zweeden AA *et al*. (2021)	33866108
ESCC	qRT‐PCR	Single	miR‐21	Increase	Serum	60	HD (60)	Diagnosis, Prognosis (OS, DFS)	Luo D *et al*. (2020)	32021418
ESCC	qRT‐PCR	Single	miR‐21	Increase	Plasma	34	HD (34)	Diagnosis	Samiei H *et al*. (2022)	34799871
EAC	ddPCR	Single	miR‐92‐3p; miR‐345‐3p	Increase	Serum	8	Barrett's esophagus (8); Intraepithelial neoplasia (12)	Diagnosis	Fassan M *et al*. (2020)	32131978
GC	qRT‐PCR	Panel	[miR‐18a, miR‐181b, miR‐335]	–	Serum	795	HD (370)	Diagnosis	Izumi D *et al*. (2021)	34427680
GC	qRT‐PCR	Single	miR‐92a‐3p	Decrease	EV (Serum)	131	HD (122)	Diagnosis	Lu X *et al*. (2021)	33533649
GC	qRT‐PCR	Single	miR‐17; miR‐25; miR‐133b	–	Serum	120	HD (102)	Diagnosis	ZiaSarabi P *et al*. (2019)	30861177
GC	qRT‐PCR	Single	miR‐1246	Increase	EV (Serum)	117	HD (82); Benign gastric disease (30)	Diagnosis	Shi Y *et al*. (2020)	31506750
GC	qRT‐PCR	Single	miR‐421	Increase	Plasma	90	HD (45); Precancerous lesions (89)	Diagnosis	Chen J *et al*. (2019)	31245174
GC	qRT‐PCR	Single	miR‐650	Increase	Plasma	90	HD (45); Precancerous lesions (90)	Diagnosis	Chen J *et al*. (2020)	32994817
GC	NGS‐based, qRT‐PCR	Panel	[miR‐1307‐3p, piR‐019308, piR‐004918, piR‐018569]	–	Serum	70	HD (60)	Diagnosis	Ge L *et al*. (2020)	33169519
GC	qRT‐PCR	Single	miR‐25	Increase	Serum	56	HD (78)	Diagnosis, Prognosis (OS)	Kong Y *et al*. (2019)	30909187
GC	NGS‐based	Single	miR‐1290	Increase	Serum	50	HD (50)	Diagnosis	Xu L *et al*. (2021)	34479528
GC	qRT‐PCR	Single	miR‐204; miR‐182	Increase	Serum	35	HD (25); *Helicobacter pylori*‐gastric ulcer (40)	Diagnosis	Mohamed WA *et al*. (2019)	30945348
HCC	qRT‐PCR	Single	miR‐30a; miR‐122; miR‐125b; miR‐200a; miR‐374b; miR‐15b; miR‐107; miR‐320b; miR‐645	–	Plasma	349	None	Prognosis (OS), Treatment (Regorafenib)	Teufel M *et al*. (2019)	30738047
HCC	qRT‐PCR	Single	let‐7c	Increase	Plasma	140	None	Prognosis (DFS), Recurrence	Canale M *et al*. (2022)	34366240
HCC	Microarray, qRT‐PCR	Panel	[miR‐122‐5p, miR‐453‐3p]	–	Serum	127	HD (20); Type 2 Diabetes (1888); Other cancer with type 2 diabetes (487)	Diagnosis	Lee HM *et al*. (2021)	34887498
HCC	Microarray, qRT‐PCR	Single	miR‐3185; miR‐4507	Decrease	Serum	122	None	Prognosis (OS)	Pascut D *et al*. (2020)	33144628
HCC	qRT‐PCR	Single	miR‐1246	Increase	Serum	121	HD (15); Other liver diseases (73)	Diagnosis, Prognosis (OS), Recurrence	Chuma M *et al*. (2019)	30920086
HCC	qRT‐PCR	Single	miR‐200c‐3p; miR‐222‐5p; miR‐512‐3p	–	Plasma	117	None	Prognosis (OS), Treatment (Sorafenib)	de la Cruz‐Ojeda P *et al*. (2022)	36078082
HCC	qRT‐PCR	Single	miR‐320a	Decrease	EV (Serum)	104	HD (50); Chronic liver disease (55)	Diagnosis, Prognosis (OS)	Hao X *et al*. (2020)	32339037
HCC	qRT‐PCR	Single; Panel	miR‐16; [miR‐16, miR‐122]	Increase	Serum	100	Liver cirrhosis (20); HD (20); Other liver disease (80)	Diagnosis	Fang Y *et al*. (2022)	35666610
HCC	qRT‐PCR	Single	miR‐518‐5p	Increase	Serum	100	HD (8); Liver cirrhosis (11)	Diagnosis, Prognosis (OS), Treatment (Sorafenib)	Fernández‐Tussy P *et al*. (2021)	34050139
HCC	qRT‐PCR	Single	miR‐122	Increase	Plasma	96	Liver cirrhosis (55); Chronic hepatitis (98)	Diagnosis	Trung NT *et al*. (2020)	32424223
CC	Other	Panel	[miR‐21, miR‐122]	–	Plasma	359	HD (204); Benign liver lesion (243); Other liver malignancies (195)	Diagnosis	Hu J *et al*. (2021)	33805513
CC + GBC	NGS‐based, qRT‐PCR	Panel	[let‐7a‐3p, miR‐92b‐5p, miR‐145‐3p, miR‐582‐3p]	–	Blood	218	HD (99); Other diseases [pancreatic cancer; pancreatitis; pancreatic benign tumor] (69)	Diagnosis	Hogdall D *et al*. (2022)	35750139
CC	qRT‐PCR	Panel	[miR‐122, miR‐192, miR‐29b, miR‐155]	–	Serum	94	HD (40)	Diagnosis, Prognosis (OS), Recurrence	Loosen SH *et al*. (2019)	30653586
GBC	qRT‐PCR	Panel	[miR‐182, miR‐21, miR‐1, miR‐130, miR‐146]	–	Serum	63	HD (21); Cholecystitis (19)	Diagnosis	Srivastava P *et al*. (2023)	36682281
CC	qRT‐PCR	Single	miR‐150‐5p	Decrease	Serum	35	HD (35)	Diagnosis	Salem PES *et al*. (2020)	33161598
PC	ddPCR	Single	miR‐1290	Increase	Plasma	167	HD (267)	Diagnosis	Tavano F *et al*. (2018)	30401891
PC	Microarray, ddPCR	Single	miR‐1273 g‐3p, miR‐122‐5p	–	Plasma	132	HD (170); IPMN (10); Chronic pancreatitis (10)	Diagnosis	Mazza T *et al*. (2020)	32117716
PC	qRT‐PCR	Single; Panel	miR‐19b‐3p; [let‐7b‐5p, miR‐192‐5p, miR‐19a‐3p, miR‐19b‐3p, miR‐223‐3p, miR‐25‐3p]	Increase	Serum	129	HD (107)	Prognosis (OS); Diagnosis	Zou X *et al*. (2019)	31006985
PC	qRT‐PCR	Single	miR‐499a‐5p	Increase	Serum	124	HD (100); Benign pancreatic disease (100)	Diagnosis	Shi Q *et al*. (2019)	31710444
PC	qRT‐PCR	Single; Panel	miR‐33a‐3p; miR‐21‐5p; miR‐320a; miR‐93‐5p; [miR‐33a‐3p, miR‐320a]	Increase	Plasma	116	HD (51); Chronic pancreatitis (23)	Diagnosis	Vila‐Navarro E *et al*. (2019)	31009404
PC	Microarray, qRT‐PCR	Panel	[miR‐130a‐3p, miR‐1228, miR‐21‐5p, miR‐223‐3p, miR‐7975, miR‐8069]	–	EV (Plasma)	92	HD (85); Chronic pancreatitis (39); Other pancreatic tumors (46)	Diagnosis	Yang G *et al*. (2023)	36357994
PC	Other	Panel	[miR‐10b, let‐7a]	–	Plasma	90	HD (60); Chronic pancreatitis (35)	Diagnosis, Recurrence	Masterson AN *et al*. (2023)	36853001
PC	NGS‐based	Single	miR‐1290	Increase	Serum	46	HD (50)	Diagnosis	Xu L *et al*. (2021)	34479528
PC	qRT‐PCR	Single	miR‐21; miR‐210	Increase	EV (Serum)	30	Chronic pancreatitis (10)	Diagnosis	Wu L *et al*. (2020)	32724386
PC	ddPCR	Single	miR‐1307	Increase	Plasma	18	None	Prognosis (OS), Treatment (FOLFIRINOX)	Carotenuto P *et al*. (2021)	34795259
CRC	qRT‐PCR	Single	miR‐449a	Decrease	Plasma	343	HD (162)	Diagnosis, Prognosis (OS)	Fu D *et al*. (2021)	33847612
CRC	Microarray, qRT‐PCR	Panel	[miR‐100, miR‐92a, miR‐16, miR‐30e, miR‐144‐5p, let‐7i]	–	EV (Plasma)	210	None	Treatment (Oxaliplatin‐based chemo)	Han J *et al*. (2020)	33072545
CRC	qRT‐PCR	Panel	[let‐7g‐5p, miR‐19a‐3p, miR‐23a‐3p, miR‐92a‐3p, miR‐144‐5p, miR‐21‐5p, miR‐27a‐3p]	–	Serum	198	HD (178)	Diagnosis	Raut JR *et al*. (2021)	34376648
CRC	qRT‐PCR	Panel	[miR‐377‐3p, miR‐381‐3p]	–	EV (Serum)	175	HD (171)	Diagnosis	Wang L *et al*. (2022)	34854318
CRC	qRT‐PCR	Panel	[miR‐193a‐5p, miR‐210, miR‐513a‐5p, miR‐628‐3p]	–	Plasma	149	HD (110)	Diagnosis, Recurrence	Nakamura K *et al*. (2022)	35850198
CRC	qRT‐PCR	Single	miR‐30a‐5p	Decrease	Serum	138	HD (60); Benign lesion (50)	Diagnosis, Prognosis (OS)	Sun Y *et al*. (2019)	30829615
CRC	NGS‐based, qRT‐PCR	Single	miR‐320c	Increase	EV (Plasma)	120	HD (129)	Diagnosis, Prognosis (OS, DFS), Recurrence, Treatment	Yang CK *et al*. (2022)	35849310
CRC	qRT‐PCR	Single	miR‐98	Decrease	Serum	115	HD (50)	Diagnosis, Prognosis (OS, DFS)	Wang YG *et al*. (2019)	31646564
CRC	Microarray, qRT‐PCR	Single	miR‐4442	Increase	Plasma	108	None	Prognosis (RFS), Recurrence	Shibamoto J *et al*. (2023)	37510319
CRC	qRT‐PCR	Single	miR‐21	Increase	Plasma	90	None	Prognosis (OS)	Varkaris A *et al*. (2019)	30636774

CC, cholangiocarcinoma; cfmiR, cell‐free miR; CRC, colorectal cancer; ddPCR, droplet digital polymerase chain reaction; DFS, disease‐free survival; EAD, esophageal adenocarcinoma; EC, esophageal cancer; ESCC, esophageal squamous cell carcinoma; EV, extracellular vesicles; GBC, gallbladder cancer; GC, gastric cancer; HCC, hepatocellular carcinoma; HD, healthy donor; NGS, next‐generation sequencing; OS, overall survival; PC, pancreatic cancer; PFS, progression‐free survival; PMID, PubMed identification; qRT‐PCR, quantitative reverse transcription polymerase chain reaction.

One challenge in EC is the existence of two different major histological subtypes. Due to the differences in their biological characteristics, there is a significant possibility of variations in cfmiR profiles. Therefore, pursuing the specific cfmiR signatures of each EC subtype may complement tissue biopsy and improve diagnosis. Also, defining neoadjuvant/adjuvant treatment response‐related cfmiRs may help to monitor treatment EC.

#### 4.2.3 Gastric cancer

Gastric cancer (GC) is the fifth most commonly diagnosed cancer, and the fourth leading cause of cancer‐associated mortality worldwide [27]. It is generally recognized that factors such as diet, alcohol consumption, *Helicobacter pylori*, and Epstein–Barr virus infection have impacts on increasing the risk of GC development [70]. To demonstrate the effectiveness of cfmiRs for the early detection and diagnosis of GC, Izumi *et al*. [71] conducted a large‐scale, multicenter validation study using The Cancer Genome Atlas database. Subsequently, the authors refined the miR signature using two different public datasets and validated the obtained candidates with retrospective serum samples from 504 GC patients and 115 HD matched cohorts. Ultimately, a 3‐miR panel (miR‐18a, miR‐181b, and miR‐335) showed high diagnostic performance (training cohort, AUC = 0.87; validation cohort, AUC = 0.87).

Among the studies reviewed, only Kong *et al*. [72] investigated the prognostic significance of miR‐25. They measured the levels of miR‐25 in plasma samples from 184 GC patients and showed that high miR‐25 levels are associated with poor prognosis, lymph node metastasis, and depth of tissue invasion. Furthermore, multivariate analysis showed that elevated levels of miR‐25 in serum represent an independent adverse prognostic factor for OS (HR = 1.84, *P* = 0.037). All the other articles focused on evaluating the diagnostic value of cfmiRs [73, 74, 75, 76, 77, 78, 79, 80] and are summarized in Table 3.

These studies on GC have demonstrated the significance of cfmiRs as novel biomarkers. Considering that most of these studies utilized serum, a meta‐analysis of the studies could determine common cfmiR signatures and performance. In addition, GC is generally suggested to be significantly associated with *Helicobacter pylori* infection [81]. Specific cancer‐related signaling pathways and the expression of immune and inflammation‐related genes have been reported to be regulated by several miRs associated with *Helicobacter pylori* infection in cancer cells and tissues. However, the detection of such miRs in the biofluid circulation has not yet been sufficiently validated [81]. Finally, there is a need for more studies assessing prognostication and monitoring treatment in GC patients.

#### 4.2.4 Hepatocellular carcinoma

Hepatocellular carcinoma (HCC) is the most common primary cancer of the liver and accounts for 90% of hepatic cancers [82]. HCC ranks as the sixth most prevalent neoplasm worldwide and is the third leading cause of cancer‐related deaths [27]. HCC occurrence is associated with the presence of chronic liver diseases and related risk factors such as hepatitis B or C viruses (viral hepatitis), alcohol consumption (alcoholic hepatitis), diabetes, and obesity (nonalcoholic hepatitis) [82]. Due to the multiple causes associated with HCC development, better noninvasive blood assays are needed for early detection.

Lee *et al*. [83] proposed two miRs (miR‐122‐5p and miR‐455‐3p) with potential diagnostic value using prediagnostic serum from 2561 patients with type 2 diabetes, including 127 individuals diagnosed with liver cancer. Patients with liver cancer showed that miR‐122‐5p and miR‐455‐3p were higher in serum levels 0–4 years before the diagnosis compared to noncarrying individuals (AUC = 0.77). Furthermore, as an assessment of risk association, the study revealed that a one‐unit increase in miR‐455‐3p serum levels was associated with an odds ratio increase of 1.021–1.022.

Pascut *et al*. [84] investigated the prognostic role of cfmiRs in liver cancer and reported an exploration of cfmiR biomarkers based on biological network analysis techniques. They identified miR modules as candidate biomarkers using weighted gene coexpression network analysis and validated them using qRT‐PCR on serum samples from 102 HCC patients. Decreased levels of miR‐4507 and miR‐3185 before the start of treatment were associated with shorter OS (HR = 0.35, *P* = 0.034; HR = 0.41, *P* = 0.0001, respectively).

Two studies focused on dynamic changes in specific miRs, such as miR‐1246 and let‐7c, as biomarkers for early recurrence of HCC [85, 86]. These studies suggest that the continuous detection of miRs in the blood before and after treatment reflects HCC tumor volume. However, due to the low reproducibility of measurement accuracy, further validation is deemed necessary for practical clinical applications in the future. Fernandez‐Tussy *et al*. [87] investigated cfmiRs for monitoring treatment in HCC and reported an association between miR‐518d‐5p and sorafenib resistance. High levels of miR‐518d‐5p in the serum of 100 HCC patients were associated with shorter treatment duration (4.81 *vs* 7.84 months, *P* = 0.006) and shorter OS (6.49 *vs* 12.39 months, *P* = 0.033). In the same study, *in vitro* experiments showed that overexpression of miR‐518d‐5p promotes sorafenib resistance. Overall, these results suggested the potential of miR‐518d‐5p as a response biomarker for sorafenib treatment.

The details of all the articles on HCC are summarized in Table 3 [88, 89, 90, 91, 92]. Due to the HCC heterogeneity and the diversity of HCC pathogenesis, the establishment of common biomarkers and molecular biological profiles remains challenging. Among the tumor types reviewed in this study, HCC exhibits the most diversified clinical applications for cfmiRs.

#### 4.2.5 Cholangiocarcinoma

Cholangiocarcinoma (CC) is a comparatively uncommon, and highly heterogenous tumor originating from the bile ducts. CC often progresses without presenting symptoms, and meanwhile, there is still no gold standard tumor marker with both high specificity and sensitivity, posing ongoing challenges in its diagnosis [61]. The five research articles reviewed focused on the diagnostic value of cfmiRs [93, 94, 95, 96, 97]. Hogdall *et al*. [93] conducted miR profiling using miR sequencing and qRT‐PCR on blood samples from 218 patients with CC. Hogdall *et al*. created two indices based on a 4‐miR panel for CC diagnosis that achieved high diagnostic performance when combined with carbohydrate antigen 19‐9 (CA19‐9) (AUC = 0.95 and 0.93, respectively).

As the only study demonstrating a prognostic role, Loosen *et al*. [95] measured serum miR levels using PCR‐based assays in 94 patients with CC who underwent surgical resection. A decrease in postoperative serum miR‐122 levels was associated with favorable OS (HR = 5.552, *P* = 0.019). In summary, the main problem with CC remains in the different subtypes, often complicated with mixed subtypes, preoperative neoadjuvant treatments, and the rareness of the disease, which limits the sample availability and well‐defined cohorts.

#### 4.2.6 Pancreatic cancer

Pancreatic cancer (PC) is one of the most lethal malignancies, with extremely poor prognosis when diagnosed in the later clinical stages, as the 5‐year survival rate remains below 10% [98]. More than half of the patients with PC have metastatic disease at the time of diagnosis [99]. Reliable biomarkers for PC are still lacking; thus, there is a need for the development of high‐precision molecular blood biopsy techniques, including cfmiRs [100]. Due to the limited number of studies, the focus was largely on diagnostic and prognostic cfmiRs for PC [79, 101, 102, 103, 104, 105, 106, 107, 108]. Two studies consistently focused on miR‐1290 [79, 104]. Tavano *et al*. [104] measured the plasma levels of miR‐1290 in 167 PC patients and 267 HD using ddPCR. The miR‐1290 showed a high diagnostic performance (AUC = 0.734) that further improved when combined with CA19‐9 (AUC = 0.914). Three reports showed the role of cfmiRs (miR‐1307, miR‐19b‐3p, and miR‐122‐5p) in prognostication [102, 108, 109]. Carotenuto *et al*. [109] evaluated miR‐1307 in plasma samples from PC patients undergoing oxaliplatin‐based chemotherapy. High levels of miR‐1307 were associated with poor OS (*P* = 0.02), suggesting the prognostic significance of cfmiRs and potential relevance associated with treatment response in PC.

Although >85% of PC have *KRAS* mutations, cfDNA diagnosis for *KRAS* has not yet reached a reliable and specific level as other cancers [110]. The studies reviewed suggested that cfmiRs do not degrade rapidly [111], are detectable reproducibly, and therefore, can be a potential reliable biomarker in PC.

### 4.3 Primary brain tumors

#### 4.3.1 Glioma

Gliomas are the most common frequent tumors of the central nervous system in adults [112]. The most challenging issue is early detection. Tissue biopsies have significant complications and imaging can be misleading. In the six articles reviewed, the same cfmiRs were found to have multiple clinical utilities (Table 4) [113, 114, 115, 116, 117, 118]. Wang *et al*. [118] demonstrated the potential diagnostic significance of single serum miR‐214 levels in 100 glioma patients before surgical resection using qRT‐PCR assay (AUC = 0.885). The authors also showed that high miR‐214 levels represent an independent prognostic factor (HR = 14.96, *P* = 0.016). By measuring paired serum samples before and after treatment, miR‐214 levels correlated with changes in treatment response (*P* < 0.001), suggesting a potential application in monitoring recurrence and/or treatment. Morokoff *et al*. [115] conducted miR sequencing on serum samples from a cohort of 91 glioma patients. The cfmiR profiles were analyzed using machine‐learning algorithms to create an accurate 9‐cfmiR diagnostic panel (AUC = 0.998).

**Table 4:** Summary of the cfmiRs with potential clinical utility for brain tumors.

Tumor type	Technology	cfmiR signature		cfmiR status	Analyte	Patients (*N*)	Control group (*N*)	Clinical variable	Author (year)	PMID
GBM	qRT‐PCR	Single	miR‐100	Decrease	Serum	95	HD (60)	Diagnosis, Prognosis (OS)	Zhang H *et al*. (2019)	30530966
GBM	qRT‐PCR	Single	miR‐21; miR‐222; miR‐124‐3p	Increase	EV (Serum)	52	None	Prognosis (OS, DFS), Recurrence	Olioso D *et al*. (2021)	34203979
GBM	NGS‐based	Panel	[miR‐5739, miR‐3180‐3p, 1909‐3p, miR‐6075, miR‐2116‐3p, miR‐4654, miR‐6085, miR‐1287‐5p, miR‐4707‐5p, miR‐3175, miR‐4706, miR‐1180‐3p, miR‐193a‐3p, miR‐1247‐3p, miR‐4685‐3p, miR‐1202, miR‐1273e, miR‐196a‐3p]	Increase	Plasma	45	HD (73)	Diagnosis	Bustos *et al*. (2022)	35013528
GBM	NGS‐based	Panel	[miR‐5739, miR‐3180‐3p]	Increase	Plasma	45	HD (73)	Recurrence	Bustos *et al*. (2022)	35013528
GBM	qRT‐PCR	Single	miR‐1238	Increase	EV (Serum)	26	None	Diagnosis	Yin J *et al*. (2019)	30917935
GBM	qRT‐PCR	Single	miR‐17‐5p; miR‐125b; miR‐221	Increase	Serum	25	HD (20)	Diagnosis, Prognosis (OS, DFS), Recurrence, Treatment (Temozolomide[+radiation])	Swellam M *et al*. (2021)	32989634
GBM	qRT‐PCR	Single	miR‐210; miR‐185; miR‐449; miR‐5194	–	EV (Plasma)	25	Head trauma (15)	Diagnosis	Tabibkhooei A *et al*. (2020)	31896490
GBM	qRT‐PCR	Single	miR‐221; miR‐222	–	Serum	20	HD (20)	Diagnosis, Prognosis (DFS), Treatment	Swellam M *et al*. (2019)	31422498
GBM	qRT‐PCR	Single	miR‐21; miR‐181	–	Serum	20	HD (20)	Diagnosis, Prognosis (OS, PFS)	Labib EM *et al*. (2022)	32316783
GBM	NGS‐based	Single	miR‐486‐3p	Increase	EV (Serum)	12	HD (16); Astrocytoma (10)	Diagnosis	Hallal S *et al*. (2020)	32668808
GBM	NGS‐based	Single	miR‐433‐3p; miR‐195‐5p; miR‐29a‐3p	Decrease	Plasma	6	HD (6)	Diagnosis	Géczi D *et al*. (2021)	34064637
GL	qRT‐PCR	Single	miR‐214	Increase	Serum	100	HD (100)	Diagnosis, Prognosis (OS), Recurrence	Wang J *et al*. (2019)	30794970
GL	NGS‐based	Panel; Single	[miR‐320e, miR‐223, miR‐23a‐3p, miR‐21]; [miR‐320e, miR‐223, miR‐16‐5p, miR‐484, miR‐520a, miR‐532, miR‐630, miR‐651, miR‐761]; miR‐320e; miR‐223; miR‐21	–	Serum	91	HD (17)	Diagnosis; Recurrence	Morokoff A *et al*. (2020)	32915353
GL	NGS‐based, ddPCR	Panel	[miR‐1, miR‐26a‐1, miR‐487b]	Decrease	Serum	52	HD (15)	Diagnosis, Prognosis (OS, DFS)	Díaz Méndez AB *et al*. (2023)	36932446
GL	NGS‐based	Panel	[miR‐4714‐3p, miR‐551b, miR‐4505, miR‐6090, miR‐6089, miR‐3960, miR‐936, miR‐1207‐5p, miR‐202‐3p, miR‐3676‐5p, miR‐4634, miR‐4539, miR‐4299]	–	Serum	47	None	Prognosis (OS, DFS)	Iannó MF *et al*. (2022)	36077842
GL	qRT‐PCR	Single	miR‐210; miR‐185; miR‐449; miR‐5194	–	EV (Plasma)	25	Head trauma (15)	Diagnosis	Tabibkhooei A *et al*. (2020)	31896490
GL	qRT‐PCR	Single	miR‐454‐3p	Increase	EV (Serum)	24	HD (24)	Diagnosis, Prognosis (OS), Recurrence	Shao N *et al*. (2019)	30413650

cfmiR, cell‐free miR; ddPCR, droplet digital polymerase chain reaction; DFS, disease‐free survival; EV, extracellular vesicles; GBM, glioblastoma; GL, glioma; HD, healthy donor; NGS, next‐generation sequencing; OS, overall survival; PMID, PubMed identification; qRT‐PCR, quantitative reverse transcription polymerase chain reaction.

#### 4.3.2 Glioblastoma

Glioblastoma (GBM) is the most aggressive subtype among gliomas, corresponding to World Health Organization grade IV [119, 120]. Patients with GBM have an extremely poor prognosis, with a median survival of less than 18 months [121]. One of the major issues of cfNA analysis in the assessment of primary brain tumors is the site in the brain and the size of the lesion, which limits the release of cfNA systemically. In addition, primary brain tumors harbor a low number of mutations, which limit further successful cfNA assessment [122].

Due to the poor prognosis of GBM patients, sample collection and follow‐up are challenging; this is why most of the studies reviewed had sample sizes of 30 or fewer patients (Table 4) [117, 123, 124, 125, 126, 127, 128, 129, 130, 131]. Zhang *et al*. [131] profiled the serum of 95 GBM patients and 60 HD using qRT‐PCR. Low miR‐100 levels demonstrated high diagnostic accuracy (AUC = 0.839). Additionally, low serum levels of miR‐100 were an independent factor for poor OS (HR = 3.75, *P* = 0.007) and progression‐free survival (PFS; HR = 4.21, *P* = 0.001). Bustos *et al*. [123] profiled 2083 cfmiRs using plasma samples of 32 primary GBM and 13 recurrent GBM patients and found two miRs with the potential clinical utility to distinguish between primary and recurrent GBM from HD or low‐grade gliomas.

CfmiRs are small and can easily pass through the blood–brain barrier, offering a potential new approach for diagnosis, prognostication, monitoring recurrence, and treatment response [132]. Cerebral spinal fluid (CSF) may be an alternative to blood assays. CfmiRs have been detected in CSF and, thus, there is a need for further exploration.

### 4.4 Melanoma

Cutaneous melanoma (CM) is a tumor that arises from the malignant transformation of melanocytes and there has been an increase in incidence over the past decade in Western societies [133]. While CM tumors have aggressive biological characteristics and a high propensity for developing distant organ metastasis, CM is one of the solid tumors that show the best rate of response to immunotherapy [134, 135, 136]. Therefore, the exploration of biomarkers is crucial for understanding the systemic pathology of CM [16, 122]. One characteristic of CM is the high tumor mutation burden. Despite the extensive evidence demonstrating the potential of cfmiR, there are many challenges (such as differences in detection methods, sample processing techniques, and significant variations from cohort to cohort) that seem to affect cfmiR assessment, not only in CM, but across all solid tumors.

All 10 articles on CM focused on validating panels of multiple rather than single cfmiRs. CfmiR panels ranged from two miRs to composite signatures comprising over 30 miRs (Table 5) [131, 137, 138, 139, 140, 141, 142, 143, 144, 145]. Van Laar *et al*. [144] independently validated the previously identified a 38‐miR signature in a cohort of 583 patients, including 372 individuals with stage I–IV invasive CM. Their validation demonstrated that the 38‐miR signature exhibited high diagnostic performance (AUC = 0.98). Furthermore, the authors created a new 12‐miR signature score that was an independent prognostic factor for disease‐specific survival (HR = 7.69, *P* = 0.0011). Bustos *et al*. [137] conducted comprehensive whole transcriptome assays containing 2083 miRs on 158 plasma samples collected before and after immunotherapy from 47 patients with stage III–IV metastatic melanoma. Using machine‐learning algorithms, the authors identified nine miRs related to immune checkpoint inhibitor (ICIs) response. The identified signature was measured in samples collected before and after treatment. Among them, levels of three miRs (miR‐4649‐3p, miR‐1234‐3p, and miR‐615‐3p) were found to be associated with complete response (CR) in post‐treatment samples from stage IV patients. These miR levels showed a dynamic decrease after treatment in responders (*P* < 0.029), demonstrating the potential role in monitoring immunotherapy response.

**Table 5:** Summary of the cfmiRs with potential clinical utility for melanoma.

Tumor type	Technology	cfmiR signature		cfmiR status	Analyte	Patients (*N*)	Control group (*N*)	Clinical variable	Author (year)	PMID
CM	NGS‐based	Panel	[miR‐4532, miR‐1306‐5p, miR‐34a‐5p, miR‐301a‐3p, miR‐181b‐5p, miR‐181d‐5p, miR‐299‐3p, miR‐454‐3p, miR‐450a‐5p, miR‐1537‐3p, miR‐424‐5p, miR‐27a‐3p, miR‐152‐3p, miR‐1973, miR‐3928‐3p, miR‐1910‐5p, miR‐520d‐3p, miR‐154‐5p, miR‐205‐5p, miR‐337‐5p, miR‐4787‐3p, miR‐497‐5p, miR‐431‐5p, miR‐548a‐5p, miR‐522‐3p, miR‐624‐3p, miR‐1302, miR‐548ad‐3p, miR‐764, miR‐1‐5p, miR‐1258, miR‐1269a, miR‐3131, miR‐5481, miR‐553, miR‐2682‐5p, miR‐138‐5p, miR‐219a‐2‐3p, miR‐1264]; [miR‐122‐5p, miR‐107, miR‐125a‐5p, miR‐151a‐3p, miR‐30d‐5p, miR‐598‐3p, miR‐22‐3p, miR‐204‐5p, miR‐378i, miR‐21‐5p, miR‐4516, miR‐630]	–	Plasma	372	Benign naevi (210)	Diagnosis; Prognosis (DSS)	Van Laar R *et al*. (2023)	37144735
CM	qRT‐PCR	Panel	[miR‐532‐5p, miR‐106b]	–	EV (Serum)	150	HD (150)	Diagnosis	Tengda L *et al*. (2018)	29750752
CM	NGS‐based	Panel	[miR‐3937, miR‐1299, miR‐1273e, miR‐670‐3p, miR‐1225‐3p, miR‐574‐5p, miR‐6780b‐5p, miR‐3674, miR‐7111‐5p, miR‐1273a, miR‐6877‐5p, miR‐6775‐5p, miR‐4279, miR‐5585‐3p, miR‐548d‐5p, miR‐7114‐3p, miR‐1207‐5p, miR‐3135a, miR‐6789‐3p, miR‐1273c, miR‐1228‐3p, miR‐1273 g‐5p, miR‐143‐5p, miR‐6795‐3p, miR‐6126, miR‐920, miR‐378f, miR‐3663‐5p, miR‐3184‐3p, miR‐5694, miR‐6796‐3p, miR‐6741‐3p, miR‐4664‐3p, miR‐4665‐5p, miR‐671‐5p]	–	Plasma	80	HD (48); Glioblastoma (36); Breast cancer brain metastasis (7); Lung cancer brain metastasis (6)	Diagnosis, Treatment (anti‐PD‐1, anti‐CTLA4)	Bustos MA *et al*. (2020)	32630542
CM	qRT‐PCR	Panel	[miR‐579‐3p, miR‐4488]	–	Serum	70	None	Prognosis (PFS), Treatment (MAPK inhibitor)	Ruggiero CF *et al*. (2022)	36438490
CM	NGS‐based, qRT‐PCR	Panel	[miR‐1246, miR‐485‐3p]	–	Plasma	57	None	Prognosis (PFS), Treatment (MAPK inhibitor)	Levati L *et al*. (2022)	35954369
CM	NGS‐based	Single; Panel	miR‐615‐3p; miR‐3197; [miR‐6794‐5p, miR‐3175, miR‐615‐3p, miR‐4649‐3p, miR‐6511‐3p, miR‐4745‐3p, miR‐1234‐3p, miR‐4306, miR‐4271]	–	Plasma	47	HD (73)	Diagnosis, Recurrence, Treatment (anti‐PD‐1, anti‐CTLA4)	Bustos MA *et al*. (2020)	33202891
CM	Microarray	Single	miR‐16‐5p; miR‐17‐5p; miR‐20a‐5p	Decrease	Serum	33	None	Treatment (anti‐PD‐1)	Nakahara S *et al*. (2020)	31843231
CM	NGS‐based, qRT‐PCR	Panel	[miR‐375, miR‐215‐5p, miR‐141‐3p, miR‐200a‐3p, miR‐200b‐3p, miR‐200c‐3p]	–	EV (Plasma)	20	HD (5)	Diagnosis	Schneegans S *et al*. (2020)	32246814
UVM	Microarray	Single; Panel	miR‐204‐5p; [miR‐16, miR‐145‐5p, miR‐146a‐5p, miR‐204‐5p, miR‐211‐5p, miR‐363‐3p]	–	Serum	55	Uveal nevus (10)	Diagnosis, Prognosis (OS)	Stark MS *et al*. (2019)	31737436
UVM	NGS‐based, qRT‐PCR	Panel	[miR‐144‐5p, miR‐191‐5p, miR‐223‐3p, miR‐483‐5p, miR‐203a]	–	EV (Serum)	20	HD (20)	Diagnosis	Wróblewska JP *et al*. (2022)	36619870

cfmiR, cell‐free miR; CM, cutaneous melanoma; DSS, disease specific‐free survival; EV, extracellular vesicles; HD, healthy donor; NGS, next‐generation sequencing; OS, overall survival; PFS, progression‐free survival; PMID, PubMed identification; qRT‐PCR, quantitative reverse transcription polymerase chain reaction; UVM, uveal melanoma.

In a second study, Bustos *et al*. [138] conducted integrated cfmiR evaluations in another article focusing on melanoma brain metastasis (MBM). A total of 2083 cfmiRs were measured in plasma obtained from 60 patients with MBM and 48 HD using a similar NGS‐based whole‐transcriptome analysis. A 74‐miR panel was found in MBM compared to HD. These cfmiR signatures were further compared with cfmiR profiles obtained from the plasma of patients with LC brain metastasis, BC brain metastasis, and GBM, resulting in the identification of the 6‐miR signature (miR‐5694, miR‐6796‐3p, miR‐6741‐3p, miR‐4664‐3p, miR‐4665‐5p, miR‐671‐5p). A 35‐miR signature found in pretreatment samples from patients with brain metastasis showed an AUC of 0.911 compared to HD. The authors also included a small sample size cohort of 20 patients with metastatic melanoma to investigate potential cfmiRs. Specific signatures were found associated with ICIs response, but were also consistently detected in MBM and metastatic melanoma patients under ICIs [138].

Overall, there is a need for larger studies assessing metastatic melanoma patients receiving ICIs, as well as for longitudinal assessment. CfmiRs can also be used to monitor adverse related events, a serious clinical issue that forces patients to lower the dose or stop ICIs treatment.

### 4.5 Gynecological cancers

#### 4.5.1 Ovarian cancer

Ovarian cancer (OC) is a leading cause of death among gynecologic malignancies and is frequently asymptomatic until advanced stages [27, 146]. Early diagnosis of OC has remained a major challenge, especially due to the difficulty of detecting symptoms and the poor prognosis of advanced stages. Gahlawat *et al*. [147] evaluated the combination of plasma cfmiRs measured by qRT‐PCR with the tumor marker carbohydrate antigen 125 (CA‐125) as a potential diagnostic modality. A 7‐miR signature (miR‐92a, miR‐200c, miR‐320b, miR‐320c, miR‐335, miR‐375, miR‐486) was evaluated as diagnostic biomarkers in a cohort of 100 patients with OC, 45 with the benign ovarian disease, and 99 age‐matched HD. The combined cfmiR signature could distinguish cases of benign disease (AUC = 0.77), early‐stage OC (AUC = 0.81), and late OC (AUC = 0.9) from HD. The addition of binary CA‐125 (cutoff 35 U·mL^−1^) to the 7‐miR signature improved the diagnostic performance (benign disease, early OC, and late OC; AUC = 0.82, 0.93, and 0.96, respectively). Yoshida *et al*. [148] investigated the prognostic impact of pretreatment serum cfmiR profiles of 175 patients with high‐grade serous OC using microarrays containing 2038 miRs. High levels of miR‐187‐5p and miR‐6875 were associated with poorer OS and PFS, and miR‐6727‐5p, miR‐6850‐5p, and miR‐1908‐5p were associated only with poorer PFS. The cfmiR profiles were used to create prognostic indices for OS and PFS. The miR signature was demonstrated to serve as an independent prognostic factor in multivariate analyses (OS index, HR = 2.343, *P* = 0.015; PFS index, HR = 2.357, *P* = 0.005).

Although each study reviewed here suggested a significant role for cfmiRs in OC, there was considerable heterogeneity in the cfmiRs utilized among studies. The investigated cfmiRs were only applied to diagnosis and prognosis, with a limited usage to monitoring treatment.

#### 4.5.2 Endometrial cancer

Endometrial cancer (EnC) is the sixth most common gynecological cancer in women [27]. There exists an increasing trend of incidence in developed countries, with a higher risk associated with obesity, carcinogens, and aging [149]. Zhou *et al*. [150] performed NGS‐based cfmiR profiling and identified six miRs (miR‐106b‐5p, miR‐107, miR‐139‐3p, miR‐15a‐5p, miR‐3615, and miR‐574‐3p) from the plasma‐derived exosomal miR profiles of 25 EnC patients and age‐matched HD, as potential diagnostic biomarker candidates. The six miRs were validated in an independent cohort of 115 EnC patients. Then the authors constructed a 3‐miR panel (miR‐106b‐5p, miR‐107, and miR‐15a‐5p) showing the most significant performance (AUC = 0.873). Moreover, the integration of the 3‐miR panel into a routine tumor biomarker test (including CEA and CA125) models significantly improved the diagnostic performance (AUC = 0.90).

Only two articles of EnC were found based on this literature search criteria, which included a large number of samples size analyzed by NGS‐based assays or microarray assays. To further validate the findings and advance into the clinical application of cfmiRs as fluid molecular biopsy in EnC, a large cohort of patients are needed.

#### 4.5.3 Cervical cancer

Cervical cancer (CeC) is the fourth most common cancer among women worldwide, primarily attributed to infection with high‐risk strains of human papillomavirus (HPV) [27, 151]. It is expected that the Papanicolaou test and HPV vaccination will decrease the incidence of CeC; however, CeC remains a leading cause of cancer‐related death in women in developing countries [151]. Zheng *et al*. conducted NGS‐based miR sequencing on 121 plasma samples, including 34 cases of CeC, and identified diagnostic miR candidates. Furthermore, the findings were validated in an independent set of 203 plasma samples using ddPCR. The combination of two miRs (let‐7d‐5p and miR‐30d‐5p) demonstrated superior diagnostic performance (AUC = 0.834) to cytology testing. Among the five reviewed articles investigating the diagnostic utility of cfmiRs, no overlapping target miRs were observed (Table 6).

**Table 6:** Summary of the cfmiRs with potential clinical utility for gynecological cancers.

Tumor type	Technology	cfmiR signature		cfmiR status	Analyte	Patients (*N*)	Control group (*N*)	Clinical variable	Author (year)	PMID
OC	Microarray	Single; Panel	miR‐187‐5p; miR‐6870‐5p; miR‐1908‐5p; miR‐6727‐5p; miR‐6850‐5p; [miR‐187‐5p, miR‐6870‐5p, miR‐1908‐5p]	Increase	Serum	175	None	Prognosis (OS, PFS)	Yoshida K *et al*. (2021)	34618992
OC	qRT‐PCR	Single	miR‐622	Increase	Serum	130	None	Prognosis (OS, DFS), Recurrence, Treatment (platinum‐based chemo)	Vigneron N *et al*. (2020)	32040573
OC	qRT‐PCR	Single	miR‐34a‐5p; miR‐93‐5p	Increase	Plasma	119	None	Prognosis (OS, DFS)	Robelin P *et al*. (2020)	32712155
OC	qRT‐PCR	Single	miR‐105	Decrease	Plasma	115	None	Treatment	Li M *et al*. (2021)	33846814
OC	Microarray	Single	miR‐21; miR‐100; miR‐200b; miR‐320; miR‐16; miR‐93; miR‐126; miR‐223	–	EV (Plasma)	106	HD (29); Cystadenoma (8)	Diagnosis, Prognosis (OS)	Pan C *et al*. (2018)	30107086
OC	qRT‐PCR	Panel	[miR‐188‐3p, miR‐500a‐5p, miR‐501‐5p, miR‐501‐3p, miR‐502‐3p, miR‐532‐3p, miR‐532‐5p]	–	Plasma	100	HD (99); Benign disease (45)	Diagnosis, Prognosis (OS)	Gahlawat AW *et al*. (2022)	35931806
OC	NGS‐based	Panel	[miR‐1307‐p, miR‐450b‐5p, miR‐150‐5p, miR‐203a‐3p, miR‐29a‐3p, miR‐23b‐3p, miR‐335‐5p, miR‐32‐5p, miR‐320d, miR‐320c, miR‐1246, miR‐92a‐3p, miR‐200a‐3p, miR‐200c‐3p]	–	Serum	75	HD (100); Benign uterine mass (100)	Diagnosis	Gockley A *et al*. (2022)	35530324
OC	qRT‐PCR	Single	miR‐200c; miR‐141	–	Plasma	72	HD (53)	Diagnosis	Gahlawat AW *et al*. (2023)	37015943
OC	qRT‐PCR	Single	miR‐223	Increase	EV (Serum)	12	None	Prognosis (DFS), Recurrence	Zhu X *et al*. (2019)	30770776
OC	ddPCR	Single	miR‐320a	Increase	Serum	5	Other diseases (5)	Diagnosis	Cirillo PDR *et al*. (2020)	32703272
EnC	NGS‐based	Single; Panel	miR‐15a‐5p; [miR‐15a‐5p, miR‐106b‐5p, miR‐107]	–	EV (Plasma)	140	HD (118)	Diagnosis	Zhou L *et al*. (2021)	33781255
EnC	Microarray, qRT‐PCR	Panel	[miR‐143‐3p, miR‐195‐5p, miR‐20b‐5p, miR‐204‐5p, miR‐423‐3p, miR‐484]	–	Serum	92	HD (102)	Diagnosis	Fan X *et al*. (2021)	34076696
CeC	qRT‐PCR	Panel	[miR‐146a‐5p, miR‐151a‐3p, miR‐2110, miR‐21‐5p]	–	EV (Plasma)	97	HD (87)	Diagnosis	Ma G *et al*. (2019)	31658043
CeC	qRT‐PCR	Single	miR‐766‐5p	Increase	Serum	67	HD (67)	Diagnosis	Cai Y *et al*. (2020)	33327889
CeC	qRT‐PCR	Panel	[let‐7d‐3p, miR‐30d‐5p]	–	EV (Plasma)	63	HD (50); Intraepithelial neoplasia (90)	Diagnosis	Zheng M *et al*. (2019)	30940131
CeC	qRT‐PCR	Panel	[miR‐143, miR‐4636]	–	Serum	60	CIN (50)	Diagnosis	Yin S *et al*. (2020)	31407404
CeC	qRT‐PCR	Single	miR‐15b; miR‐34; miR‐218	–	Serum	23	HD (23); Low‐grade intraepithelial lesion (17)	Diagnosis	Ocadiz‐Delgado R *et al*. (2021)	33112434
CeC	qRT‐PCR	Single	miR‐221	Increase	EV (Serum)	20		Diagnosis	Zhou CF *et al*. (2019)	30254211

CeC, cervical cancer; cfmiR, cell‐free miR; ddPCR, droplet digital polymerase chain reaction; DFS, disease‐free survival; EnC, endometrial cancer; EV, extracellular vesicles; HD, healthy donor; NGS, next‐generation sequencing; OC, ovarian cancer; OS, overall survival; PFS, progression‐free survival; PMID, PubMed identification; qRT‐PCR, quantitative reverse transcription polymerase chain reaction.

In this section, we summarized recent findings on the research of cfmiRs in CeC. It is suggested that the E6/E7 genes expressed during the replication of the HPV virus are involved in the malignant transformation of normal cervical epithelium [151]. Several miRs, including the aforementioned let‐7d‐5p, have been reported to control these oncogenes, suggesting their potential as biomarkers [152]. However, evidence of the actual presence or detectability of these miRs in biofluids is currently limited, and further research aimed at a clinical application is increasingly anticipated. All the studies presented are summarized in Table 6, including the other reports not fully discussed in this section, but focusing on OC, EnC, and CeC [153, 154, 155, 156, 157, 158, 159, 160, 161].

### 4.6 Breast cancer (BC)

BC remains a leading cause of cancer death in women [27, 162]. Extensive research has been done to explore the role of miRs in BC diagnosis. Zou *et al*. [163] examined the serum cfmiR profiles of 183 patients diagnosed with BC and 106 HD and identified 30 dysregulated cfmiRs in BC. Furthermore, the authors constructed and optimized an 8‐miR panel composed of miR‐133a‐3p, miR‐497‐5p, miR‐24‐3p, and miR‐125b‐5p miR‐377‐3p, miR‐374c‐5p, miR‐324‐5p, and miR‐19b‐3p (AUC = 0.915) by 2‐fold cross‐validation with a total of 753 participants (including 357 BC cases). Zou *et al*. [164] measured cfmiRs in both serum and plasma samples using qRT‐PCR assays. Thereafter, the authors created a diagnostic panel in serum (miR‐188‐3p, miR‐501‐3p, miR‐502‐3p, miR‐532‐3p, and miR‐532‐5p) and plasma samples (miR‐188‐3p, miR‐500a‐5p, and miR‐501‐5p), validating their high diagnostic capabilities. Additionally, they demonstrated the correlation of miR‐188‐3p detection across samples from both panels. Shiino *et al*. [165] attempted to develop a new diagnostic method for axillary lymph node metastasis in BC as an alternative to the gold standard, sentinel lymph node biopsy, utilizing cfmiR from serum. The optimized diagnostic model combined miR‐629‐3p and miR‐4710 with several clinical‐pathological factors in serum and showed an AUC of 0.86 in the validation cohort.

Regarding the prognostic role of cfmiR in BC, Gahlawat *et al*. [166] measured the total levels of cfmiRs rather than individual cfmiRs in pretherapeutic and longitudinal plasma of 250 BC patients. The study revealed that high levels of total cfmiRs at baseline were associated with poor OS and disease‐free survival (DFS). Moreover, the multivariate analysis showed that high levels of total miRs at baseline served as an independent prognostic biomarker for BC. All the BC studies are summarized in Table 7.

**Table 7:** Summary of the cfmiRs with potential clinical utility for BC.

Tumor type	Technology	cfmiR signature		cfmiR status	Analyte	Patients (*N*)	Control group (*N*)	Clinical variable	Author (year)	PMID
BC	Microarray	Panel	[miR‐629‐3p, miR‐4710]	–	Serum	921	Benign breast disease (37)	Diagnosis	Shiino S *et al*. (2019)	30482779
BC	qRT‐PCR	Panel	[miR‐133a‐3p, miR‐497‐5p, miR‐24‐3p, miR‐125b‐5p, miR‐377‐3p, miR‐374c‐5p, miR‐324‐5p, miR‐19b‐3p]	–	Serum	504	HD (502)	Diagnosis	Zou R *et al*. (2022)	35013577
BC	qRT‐PCR	Panel	[miR‐188‐3p, miR‐500a‐5p, miR‐501‐5p]; [miR‐188‐3p, miR‐501‐3p, miR‐502‐3p, miR‐532‐3p, miR‐532‐5p]	Increase	Plasma	354	HD (404)	Diagnosis	Zou X *et al*. (2020)	31493506
BC	Microarray, ddPCR	Single	miR‐923	Increase	Plasma	253	HD (10)	Prognosis (DFS)	Lasham A *et al*. (2020)	31607655
BC	–	–	Total miR amount	Increase	Plasma	250	None	Prognosis (OS), Recurrence	Gahlawat AW *et al*. (2022)	35318434
BC	NGS‐based, qRT‐PCR	Single	miR‐423‐5p	Increase	Serum	224	HD (113)	Diagnosis	Liu D *et al*. (2021)	33493140
BC	Microarray, qRT‐PCR	Panel	[let‐7b‐5p, miR‐106a‐5p, miR‐19a‐3p, miR‐19b‐3p, miR‐20a‐5p, miR‐223‐3p, miR‐25‐3p, miR‐425‐5p, miR‐451a, miR‐92a‐3p, miR‐93‐5p, miR‐16‐5p]	–	Serum	216	HD (214)	Diagnosis	Zou X *et al*. (2021)	32894240
BC	qRT‐PCR	Single	miR‐373	Increase	Serum	196	HD (49); Benign breast disease (76)	Diagnosis	Bakr NM *et al*. (2021)	34089425
BC	qRT‐PCR	Single	miR‐99a‐5p	Increase	Plasma	194	HD (183)	Diagnosis	Garrido‐Cano I *et al*. (2020)	33050096
BC	qRT‐PCR	Single	miR‐30b‐5p	Increase	Plasma	121	HD (123)	Diagnosis	Adam‐Artigues A *et al*. (2021)	33477007

BC, breast cancer; cfmiR, cell‐free miR; ddPCR, droplet digital polymerase chain reaction; DFS, disease‐free survival; EV, extracellular vesicles; HD, healthy donor; NGS, next‐generation sequencing; OS, overall survival; PMID, PubMed identification; qRT‐PCR, quantitative reverse transcription polymerase chain reaction.

### 4.7 Lung cancer

Lung cancer (LC) is the leading cause of cancer death worldwide, and nonsmall cell lung cancer (NSCLC) accounts for ~85% of LC cases [27]. Patients with NSCLC have a poor prognosis, with a 5‐year OS rate of less than 20%. Tissue biopsy procedures for LC patients are invasive and of high risk. Thus, there has been extensive research exploring cfmiRs as a less invasive method for early diagnosis, prognosis, and treatment prediction in NSCLC (Table 8) [167, 168, 169, 170, 171, 172, 173, 174, 175, 176]. Ying *et al*. [171] identified a 5‐miR panel (let‐7a‐5p, miR‐1‐3p, miR‐1291, miR‐214‐3p, and miR‐375) with a potential role in early detection of NSCLC. The cfmiR panel was validated across multiple cohorts, confirming high performance for the diagnosis of early‐stage LC (AUC = 0.886–0.976). In another study, Fehlmann *et al*. [168] examined genome‐wide cfmiR profiles in a large cohort of 3102 participants including 606 NSCLC patients, and constructed respective 14‐miR and 15‐miR signatures that were tested in various scenarios (LC *vs* non‐LC control, LC *vs* nontumor lung diseases, or early‐stage LC *vs* non‐LC) showing high specificities and sensitivities.

**Table 8:** Summary of the cfmiRs with potential clinical utility for LC.

Tumor type	Technology	cfmiR signature		cfmiR status	Analyte	Patients (*N*)	Control group (*N*)	Clinical variable	Author (year)	PMID
LC	qRT‐PCR	Single	miR‐200b; miR‐200c	–	Plasma	2919	HD (7904)	Diagnosis	Wang YZ *et al*. (2021)	34661272
LC	Microarray	Panel	[miR‐1268b, miR‐6075]	Increase	Serum	1566	HD (1998)	Diagnosis	Asakura K *et al*. (2020)	32193503
NSCLC	qRT‐PCR	Panel	[let‐7a‐5p, miR‐1‐3p, miR‐1291, miR‐214‐3p, miR‐375]	Increase	Serum	744	HD (944)	Diagnosis	Ying L *et al*. (2020)	32943537
LC	Microarray	Panel	[miR‐1283‐3p, miR‐205‐5p, miR‐1260a, miR‐1260b, miR‐3152‐3p, miR‐378b, miR‐1202, miR‐139‐5p, miR‐16‐2‐3p, miR‐18a‐3p, miR‐23b‐3p, miR‐3907, miR‐551b‐3p, miR‐93‐3p]; [miR‐1283‐3p, miR‐205‐5p, miR‐17‐3p, miR‐1202, let‐7 g‐3p, miR‐193a‐5p, miR‐21‐3p, miR‐3610, miR‐4282, miR‐4286, miR‐452‐3p, miR‐516a‐3p, miR‐572, miR‐625‐5p]; [miR‐1283‐3p, miR‐205‐5p, miR‐1260a, miR‐1260b, miR‐3152‐3p, miR‐378b, miR‐17‐3p, miR‐564, miR‐374b‐5p]	–	Blood	606	Unaffected control (964); Non‐tumor lung disease (593); Diseases not affecting the lung (883)	Diagnosis	Fehlmann T *et al*. (2020)	32134442
NSCLC	Microarray, qRT‐PCR	Panel	[miR‐20b‐5p, miR‐3187‐5p]	Increase	EV (Serum)	276	HD (282)	Diagnosis	Zhang ZJ *et al*. (2020)	32741216
NSCLC	NGS‐based	Panel	[miR‐21‐5p, miR‐429, miR‐3679‐5p, miR‐100‐5p, miR‐141‐3p, miR‐200c‐3p, miR‐200a‐3p, miR‐2110, miR‐130‐3p, miR‐378a‐3p, miR‐98‐5p, miR‐203a, miR‐382‐5p, miR‐4532, miR‐664a‐5p, miR‐145‐3p, miR‐483‐3p, miR‐181a‐5p, miR‐1301‐3p, miR‐320e, let‐7f‐5p, miR‐424‐5p, miR‐320b, miR‐101‐3p, miR‐378c, miR‐22‐3p, let‐7a‐5p, miR‐1246, miR‐320d, miR‐32‐5p, miR‐628‐3p, miR‐190a‐5p, miR‐20b‐5p, miR‐224‐5p, miR‐139‐5p, miR‐584‐5p, miR‐369‐5p, miR‐148a‐3p, miR‐320c, miR‐3605‐3p, miR‐95‐3p, miR‐205‐5p, miR‐145‐5p, miR‐625‐3p, miR‐150‐5p]	–	Serum	213	None	Treatment (anti‐PD‐1)	Zhang Y *et al*. (2022)	36198243
NSCLC	NGS‐based	Single	miR‐425‐3p	Increase	EV (Serum)	203	None	Prognosis (PFS), Treatment (Chemotherapy)	Yuwen D *et al*. (2019)	30228154
NSCLC	qRT‐PCR	Single	miR‐185	Decrease	Serum	196	HD (80); Non‐malignant lung disease (25)	Diagnosis, Prognosis (OS, RFS)	Liu J *et al*. (2020)	33251978
NSCLC	qRT‐PCR	Single	miR‐17‐5p	Increase	EV (Serum)	172	HD (137)	Diagnosis	Zhang Y *et al*. (2019)	31146974
NSCLC	Microarray, qRT‐PCR	Panel	[miR‐520‐3p, miR‐1274b]	Increase	Serum	159	HD (120); Benign nodule (31)	Diagnosis	Zhong Y *et al*. (2021)	33340250

cfmiR, cell‐free miR; EV, extracellular vesicles; HD, healthy donors; LC, lung cancer; NGS, next‐generation sequencing; NSCLC, non‐small cell lung cancer; OS, overall survival; PFS, progression‐free survival; PMID, PubMed identification; qRT‐PCR, quantitative reverse transcription polymerase chain reaction; RFS, relapse‐free survival.

One of the frequently encountered diagnostic challenges is obtaining a preoperative diagnosis of a solitary pulmonary nodule (SPN) identified on a computed tomography (CT) scan. The main problem is that SPNs are difficult to distinguish from cancer using imaging approaches [177]. Also, lung biopsies are invasive and can be difficult to perform. Patients with SPN are recommended to undergo surveillance with repetitive CT scans for years, often without a definitive diagnosis of the SPN. To address this clinical problem, Zhong *et al*. [176] identified a significant upregulation of miR‐520c‐3p and miR‐1274b in patients with NSCLC compared to patients with benign SPN or HD. Both miR‐520c‐3p and miR‐1274b showed good performance as classifiers to distinguish early‐stage LC and SPN (AUC = 0.845, *P* < 0.0001). In a large‐scale screening cohort, Wang *et al*. [170] indicated the potential utility of miR‐200b and miR‐200c in combination with an imaging‐based diagnosis for SPN. Other reports indicated that low miR‐425‐3p levels in circulating EVs and elevated miR‐185 levels in serum were significantly associated with poor prognosis in LC [169, 172]. In addition, both miR‐425‐3p and miR‐185 correlated with chemotherapy or CRT responsiveness [169, 172]. There have been unique reports in recent years comparing the association of cfmiR levels and the response to ICIs. Zhang *et al*. [173] constructed a machine‐learning model based on 45 cfmiRs identified through NGS‐based miR profiling and clinical information for NSCLC patients treated with monotherapy of PD‐1 inhibitors. The 45‐cfmiR signature was optimized to identify responders and achieved higher performance compared to the conventional companion diagnostic and PD‐L1 assessment.

## 5 Conclusion and Future Perspectives

CfmiRs can represent promising biomarkers for diagnosis, prognosis, and monitoring of tumor progression with abundant applications across multiple diseases arising from different organ sites and of different histological subtypes. However, due to complexity, the utility of single cfmiR markers is inefficient, highlighting that a number of cfmiRs should be considered when developing biomarker panels. Investigators will need to use deep‐learning algorithms and large datasets to identify the best combinational set of cfmiRs that best adjust to the clinical utility and the tumor type, without losing sensitivity or specificity.

A limitation for cfmiRs clinical applicability may stem from the fact that cfmiRs can reflect not only the tumor cells but other cell types of the tumor microenvironment. For example, many malignant tumors induce metabolic or nutritional disorders like cachexia. Thus, the physiological changes in the tumor‐bearing host could potentially lead to alterations of cfmiRs originating from normal tissues that could globally affect the cfmiR profiles [178]. Similarly, comorbidities (cardiovascular disease, pulmonary disease, diabetes, arthritis, depression, hypothyroidism, menopause, osteoporosis, osteopenia, etc.) in cancer patients need to be considered, as they can influence the cfmiR profiles [179, 180, 181, 182, 183, 184]. Also, there is a need for characterization of the immune cell populations that may be contributing to plasma and serum cfmiR profiles, as previously discussed [185].

One of the frequent questions that arises when performing cfmiR analysis is the correlation of the cfmiRs with the miR expression levels in the corresponding tumor tissues [7]. The inclusion of paired tissue samples needs to be considered in prospective studies, as the majority of previous studies lack paired tumor tissue analysis. This is due to several factors, including, but not limited to, the amount of tissue available, the input required for downstream analysis, the resectability of certain tumors, and the quality and preservation status of the tumor tissue samples.

Another major challenge in reaching a clinical application relates to inadequate methodological reporting. The National Institutes of Health (NIH) extracellular RNA communication consortium has developed a vision for a cfmiR assay [186]. Namely, standardization strategies are needed for several detailed handling processes to improve reliability and reproducibility, ranging from biofluid sources, collection and storage times and temperatures, miR isolation, detection, and quantification techniques, each of which affects cfmiR endpoints. There is also a lack of standardized cutoffs to consider a cfmiR clinically relevant. The standardization of protocol and assays will enable cross‐studies comparisons and independent data validations. Newer techniques for detecting cfmiRs are being developed, such as NGS‐based methods, which are improving the specificity, sensitivity, and robustness of the assays.

The successful clinical implementation of cfmiRs will require the identification of specific cfmiRs for each tumor type, a deep knowledge of the common miRs correlated with each tumor type, as well as the understanding of the cfmiRs derived from normal cells. In addition, the implementation will require better technologies than current PCR‐based assays, which allow for genome‐wide assessment, minimizing preanalytical steps that can interfere with cfmiRs quantification.

CfmiRs offer an alternative source to cfDNA in many cancers, particularly those cancer types with limited genomic variants. In addition, cfmiRs are more stable in biofluids and have functional biological activities. Also, cfmiR abundance and the variety of cfmiRs in body fluids may offer more efficient alternatives to early detection of cancer than cfDNA. Future large cohorts from clinical trials combined with companion assays will support the oncological implementation of cfmiRs.

## Conflict of interest

The authors declare no conflicts of interest.

## Author contributions

YH, DSBH, and MAB conceived and designed the study, YH acquired and analyzed the data, YH and MAB interpreted the data, YH, J‐CM, RIR, JAL, TGW, DSBH, and MAB drafted the original paper, DSBH, JAL, and TGW acquired fundings.
